# A systematic mapping of public health master’s and structured doctoral programs in Germany

**DOI:** 10.1186/s12909-024-05855-8

**Published:** 2024-08-13

**Authors:** Hanna Saturska, Katrina Kufer, Sara Pedron, Gesa Meyer, Karl Emmert-Fees, Michael Laxy, Anna-Janina Stephan

**Affiliations:** 1 Professorship of Public Health and Prevention, School of Medicine and Health, Technical University of Munich, Georg-Brauchle-Ring 60/62, Munich, 80992 Germany; 2 Department of Public Health and Healthcare Management, Faculty of Medicine, Ternopil National Medical University, Ternopil, 46000 Ukraine

**Keywords:** Public health, Education, Curricula, Doctoral program, Master’s program, MPH programs, Challenges of public health education, Health professionals, Systematic mapping, Germany

## Abstract

**Background:**

Well-trained public health professionals are key to addressing both global and local public health challenges of the twenty-first century. Though availability of programs has increased, the population health science (PHS) and public health (PH) higher education landscape in Germany remains scattered. To date, no comprehensive overview of programs exists.

**Objectives:**

This study aimed to map PHS and PH master’s and structured doctoral programs in Germany, including selected program characteristics, curricula and target competencies.

**Methods:**

We conducted a systematic mapping of PHS and PH programs in Germany following a prospectively registered protocol (https://doi.org/10.17605/OSF.IO/KTCBA). Relevant master’s and doctoral programs were identified by two study authors independently searching a comprehensive higher education database, which was, for doctoral programs, supplemented with a google search. For PHS programs, general characteristics were mapped and for the subset of PH programs, in-depth characteristics were extracted.

**Results:**

Overall, 75 master’s and 18 structured doctoral PHS programs were included. Of these, 23 master’s and 8 doctoral programs focused specifically on PH. The majority of PHS master’s programs awarded a Master of Science degree (55 out of 75 programs). The PH master’s program curricula offered various courses, allowing for different specializations. Courses on topics like public health, epidemiology, health systems (research) and research methods were common for the majority of the master’s programs, while courses on physical activity, behavioral science, nutrition, and mental health were offered less frequently. Structured PH doctoral programs were mainly offered by medical faculties (6 out of 8 programs) and awarded a doctorate of philosophy (Ph.D.) (6 out of 8 programs). PH doctoral programs were very heterogeneous regarding curricula, entry, and publication requirements. There was a broad geographical distribution of programs across Germany, with educational clusters in Munich, Berlin, Bielefeld and Düsseldorf.

**Conclusion:**

Germany offers a diverse landscape of PHS and PH master’s programs, but only few structured doctoral programs. The variety of mandatory courses and competencies in these programs reflect Germany’s higher education system’s answer to the evolving demands of the PH sector. This review may aid in advancing PH education both in Germany and globally.

**Supplementary Information:**

The online version contains supplementary material available at 10.1186/s12909-024-05855-8.

## Introduction and rationale

The strain on healthcare systems through the continued global increase in non-communicable diseases highlights the need for systematic global public health capacity building [1, 2]. This need has recently been further exacerbated by new infectious disease outbreaks such as the SARS-CoV-2 which caused a global pandemic [3].

A well-trained public health workforce is crucial to address these contemporary and future public health challenges of the twenty-first century [4, 5]. Preconditions for educating the next generation of public health professionals are adequate, available and attractive degree programs, offering high-quality training and specialization opportunities [6]. In this context, the German higher education system has been confronted with demands to strengthen networks between existing public health educational programs and other public health stakeholders and to increase standardization of existing public health education [7].

In Germany, public health is officially managed by the Public Health Service (Öffentlicher Gesundheitsdienst), which is organized on the federal, state and municipal levels [8]. The Public Health Service has traditionally been primarily staffed by professionals from the fields of medicine, social work, and hygiene. However, the need to further diversify the workforce, also by incorporating dedicated public health specialists, is increasingly acknowledged [9].

Since the end of the twentieth century, the number of established population health science programs offered by different academic institutions has significantly expanded in Germany [10]. In the German language “health science” (“Gesundheitswissenschaften”) and “public health” have often been used interchangeably, although sometimes with slightly distinct emphases [11]. While public health is sometimes seen as more focused on practical and policy implementation aspects and the population health service, health science is sometimes seen as more research focused [11]. Both fields, however, share an interdisciplinary character and aim at preventing diseases and improving health [12] on a population level differentiating them from fields with a clear focus on health at the individual level, such as medicine and nursing.

**Fig. 1:**
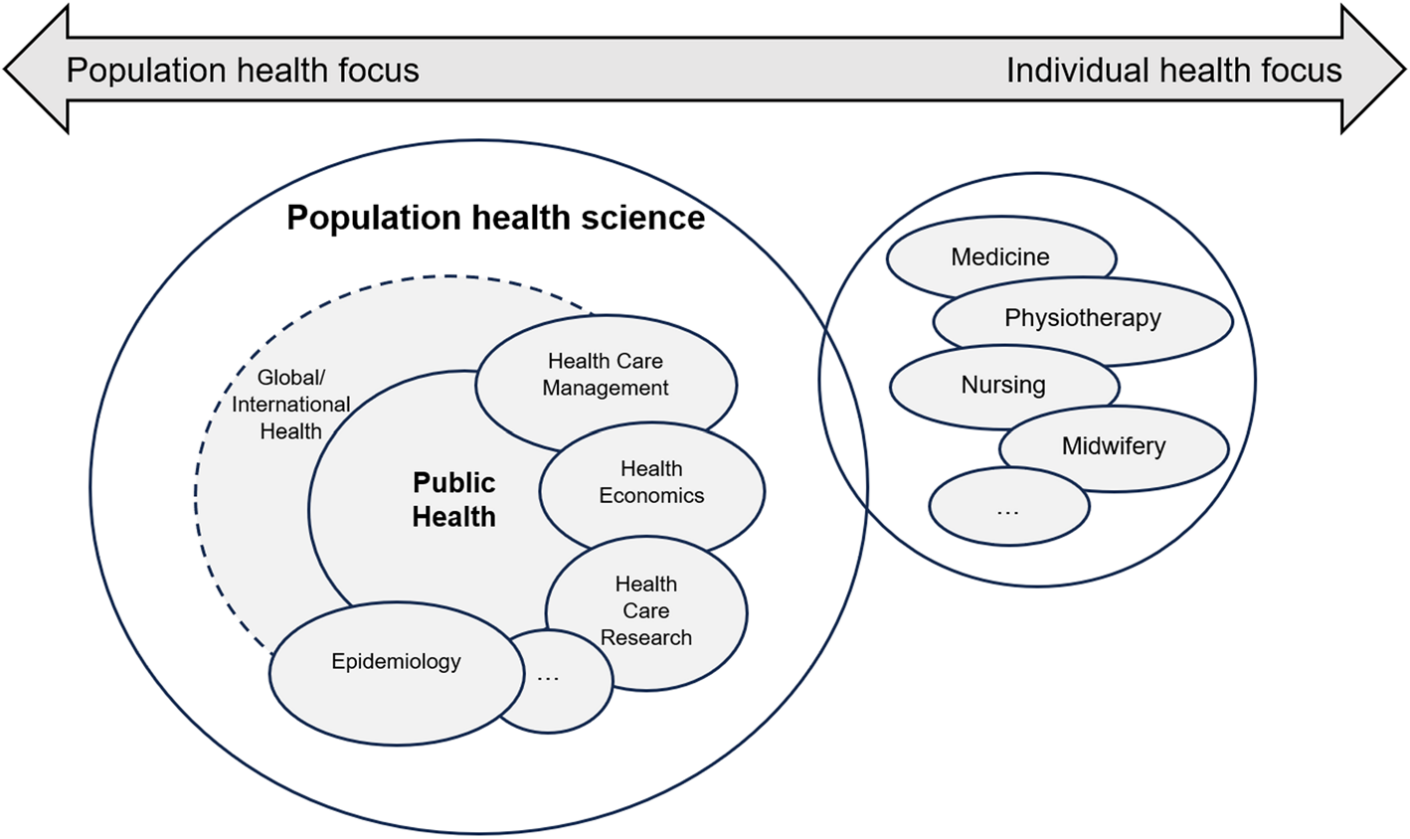
Term definitions. Note: When interpreting this figure, readers should keep in mind that it presents relationships between disciplines from a public health focused perspective and simplifies the relationships between other disciplines for the sake of readability. There are additional overlaps between disciplines, even though shown here as non-overlapping, such as between epidemiology and health economics, or medicine and midwifery. Also, depending on the context, population and individual health foci may be conceptualized not only as opposite ends on a continuum, but as mutually dependent and complementary factors

In Fig. 1, we depict our use of the following terms. We use the term “population health science” as an umbrella term for programs with interdisciplinary, research-oriented character that focus on population health. Population health sciences in our definition comprise subdisciplines including health economics, health services research and “public health”, where public health is characterized by a stronger focus on health systems, health policies, and effectiveness of population-based interventions.

Although public health master’s programs in Germany may be comparable with regard to the formal criteria required for accreditation, no subject or content specific core requirements exist [13]. Consequently, to date there is no clear core curriculum or set of core competencies at either master’s or doctoral level [7]. Even less is known about the seemingly increasing number and heterogeneous scope of population health science programs in Germany. Additional general background information on the German higher education system, is provided in Additional File 1.

In contrast the United States and United Kingdom have well-structured clear content requirements for public health education [14, 15]. In England, this has further led to the establishment of specialized training and registration options for public health professionals [15]. In Europe, efforts to overcome the existing heterogeneity and improve the quality of public health education is currently pursued by the Association of Schools of Public Health in the European Region (ASPHER) [16]. This has led to the foundation of the Agency for Accreditation in Public Health Education (APHEA), which seeks to improve credibility and transferability of qualifications, and to ensure the inclusion of core public health competencies in public health master’s programs [17]. Despite such attempts towards a more coherent scope of European higher public health education, representation of German public health programs in these networks is still low, as yet no German program has received APHEA accreditation [14]. Furthermore, only 15 public health institutions in Germany are recorded as members of ASPHER as of February 2024 [18].

In line with these international efforts, calls have been made from the German National Academy of Sciences (“Leopoldina”) and the Public Health Future Forum (“Zukunftsforum”), among others, to improve coherence of public health education, and to form better connections between educational institutions and professional public health practice in Germany [7, 10]. Achieving this, however, requires a thorough understanding of the current landscape of public health education in Germany [7].

Comprehensive mappings of public health higher education programs have previously been conducted in other geographical regions, including in South Asia [19] and Austria [20]. In the German context, a 2015 survey among 104 coordinators of bachelor’s and master’s programs provided a first overview of public health contents in those programs [21]. However, survey items focused primarily on coordinators’ individual characteristics such as professional backgrounds and their activities, such as academic networking. Additionally, the survey provided a first quantitative overview of topics taught, literature sources used for teaching purposes, and unmet resource needs perceived by teaching staff. However, by design, the study provided an aggregated and person-centered overview of the public health landscape rather than a structured mapping of individual-level program characteristics.

A longitudinal overview for Germany between 2001 and 2014 by Hartmann and colleagues found 30 master’s degree programs in health science/public health, epidemiology and health promotion in 2014, reflecting an increase in the number of programs offered since 2001 [22]. They described the programs’ general characteristics (e.g. location, degree awarded, founding year, study form and accreditation agency). The authors alluded to the heterogeneity of existing programs and called for establishing a collaborative framework among universities, to analyze and align existing module catalogues with competency profiles and to consolidate and synchronize health science related programs. However, a detailed mapping and comparison of program curricula and targeted competencies was not provided.

Furthermore, in both published overviews, no information was collected regarding doctoral programs, and to our knowledge, no other overview of German doctoral level public health education exists.

### Objective and research questions

In this study, we aimed to provide a comprehensive overview of public health education in Germany through a systematic mapping of existing population health science and public health master’s and structured doctoral programs. Our first objective was to map the landscape of master’s and doctoral level programs available in Germany in the field of population health sciences such as public health, health science, epidemiology, health economics, healthcare management, and health policy. As a second objective, based on these findings, we aimed to determine the composition of typical curricula specifically for the subset of public health master’s and structured doctoral programs to understand commonalities, differences and specializations. Third, we aimed to systematically collect information on targeted core competencies to be acquired by students within these public health master’s and doctoral programs as stated by these programs aiming to understand the intended professional perspectives for graduates (in-depth program insights).

## Methods

### Review design

This systematic mapping follows a two-stage approach based on the differentiation between “population health science” programs (which we use as umbrella term for interdisciplinary research-oriented programs with a population health focus) and “public health” programs (the subset of population health programs with a focus on health systems, health policies, and effectiveness of population-based interventions).

In a first step, we identified all population health science (PHS) master’s and doctoral programs in Germany and reported high-level information on their main structural characteristics (general PHS program mapping). In the second step, we focused specifically on the subset of all public health programs. For these public health (PH) master’s and doctoral programs, we extracted and mapped further detailed characteristics (“in-depth PH program insights”).

Within the scope of this study, we only included doctoral programs that are awarded through structured thematic programs. The heterogeneity of traditionally supervised individual German doctorates makes a systematic review in the intended format nearly impossible (for further details see Additional File 1).

As no methodological guidelines exist specifically for the systematic mapping of academic programs, we followed where appropriate, relevant guidelines for systematic literature reviews (e.g., Preferred Reporting Items for Systematic Reviews and Meta-Analyses (PRISMA) [23]). However, it should be noted that while systematic literature reviews usually comprise a quality appraisal of included studies. It was not our intention to conduct any form of subjective or objective quality assessment for the programs included in our mapping. Therefore, a quality appraisal is outside the scope of this project.

The prespecified protocol of the search and data extraction procedure can also be accessed at https://doi.org/10.17605/OSF.IO/KTCBA.

### Overall search strategy for population health science programs

#### Databases

We used the Higher Education Compass (HEC) website (www.hochschulkompass.de) as the database for identifying both master’s and doctoral population health science programs. The HEC is an official online portal from a major association of German universities (“Hochschulrektorenkonferenz”) which collects information about higher education programs in Germany published by the respective institutions [24]. The HEC website provides general information on German degrees and search fields to identify degree programs with keywords and several filter options (e.g. master’s or doctoral level) and has previously been used for similar research endeavors [21, 22].

Secondary data sources to complement information on relevant doctoral programs from the HEC were Google and the doctoral program database of the German Academic Exchange Service (DAAD) [25]. These additional databases were searched because a previous preliminary search in the HEC during protocol development suggested that, in contrast to the master’s programs, not all relevant structured doctoral programs were listed in the HEC.

#### Search strategy for master’s programs

To identify potentially relevant population health science master’s programs (general PHS program mapping), a search was conducted by consecutively entering the following search terms in the HEC program search fields: “health”, “epidemiology”, “prevention”, and their German equivalents “Gesundheit”, “Epidemiologie”, and “Prävention”. While no filters were set for mode of admission or form of study, programs were filtered by degree (master’s only).

For each search term, search results were exported into an excel table detailing program titles, institutions, and website links. Resulting excel lists from all searches were subsequently merged and duplicates removed before screening activities were initiated.

#### Search strategy for doctoral programs

A separate search for relevant population health science doctoral programs (general PHS program mapping) was conducted on the HEC website similar to the search for master’s programs, this time using the “doctoral studies” tab [24]. Doctoral programs were additionally searched for on Google and the DAAD website with the same search terms as in the HEC. References to doctoral programs, which were found during the search and screening processes for master’s programs were additionally followed up.

The Google search was performed on the respective German homepage (“www.Google.de”) using the search terms: (“Ph.D.” OR “Promotion” OR “Doctorate”) AND (“Germany” OR “Deutschland”) AND (“Gesundheit” OR “health” OR “Epidemiologie” OR “epidemiology” OR “Versorgung” OR “Prävention” OR “prevention”).

As pre-defined in the protocol, the first ten pages of Google search results were screened. Results indicating the existence of relevant population health science doctoral programs were followed up with targeted additional online searches for the respective program websites and recorded accordingly.

### Data screening

We defined separate eligibility criteria for population health science programs included in the first stage (general PHS program mapping) and the subset of public health programs included for the second stage (in-depth PH program insights). Eligibility criteria for both stages are listed in Table 1.

**Table 1:** Eligibility criteria for population health science master’s and doctoral programs (general PHS program mapping, first stage)

	Inclusion	Exclusion
Type of institution	Universities, technical universities, universities of applied science	
Level of program	Master’s, doctoral^1^	Bachelor, certificate, master’s^2^
Type of program^3^	Structured (contains courses, which are obligatory to get a degree)	Unstructured (individual, no mandatory courses)
Program focus	Public health, health(care) management/ Gesundheitsmanagement, health economics/Gesundheitsökonomie, epidemiology/Epidemiologie, health policy/Gesundheitspolitik, prevention/Prävention, health promotion/Gesundheitsförderung, international/global health, health care research/Versorgungsforschung, population health science/Gesundheitswissenschaft	Biomedical/Biomedizin, nursing or caring/Pflegewissenschaft, midwifery/Hebammenwissenschaft, physiotherapy/Physiotherapie, teaching/Lehramt, social work/Sozialarbeit, computer science/Informatik, data science/Datenwissenschaft, statistics/Statistik, sports science/ Sportwissenschaft

^1^For the screening of master’s programs the classification as “doctoral” program was an exclusion criterion

^2^For the screening of doctoral programs the classification as “master’s” program was an exclusion criterion. This specification was added after the original protocol was published for clarity

^3^This criterion was added after the original protocol was published for clarity

#### First stage eligibility – general PHS program mapping

The search results exported from the HEC and Google searches were screened for programs meeting the inclusion criteria for the general PHS program mapping. Two authors (HS, KK) screened all programs in duplicate and, in case of deviating inclusion decisions, aimed to reach consensus through discussion. If consensus was not possible a third author was involved (KEF or AJS).

#### Second stage eligibility (in-depth PH program insights)

Master’s programs meeting the first-stage eligibility criteria (general PHS program mapping) were categorized as eligible for second-stage data extraction (in-depth PH program insights) if they included the term “public health” in their title, or awarded a degree containing the term “public health” e.g., Master’s in Public Health (MPH).

For doctoral programs, inclusion for the second stage data extraction (in-depth PH program insights) based on these criteria (i.e., program or degree title) alone was deemed too restrictive. The main reason is that often multiple related disciplines can be represented under the same doctoral umbrella title (e.g. “Ph.D. in Health data sciences” or “Ph.D. in Medical Research”). Therefore, for doctoral programs, second-stage inclusion criteria were also deemed satisfied if “public health” was explicitly referenced in the program description among the types of possible projects, but not in the program title or degree awarded.

### Data extraction and variables of interest

Data extraction was conducted in duplicate by HS and KK according to predefined extraction categories. Data extraction for population health science programs meeting first stage eligibility criteria (general PHS program mapping) included the program title, institution name, institution type, institution location, form of study, duration and total of credits awarded as per the European Credit Transfer and Accumulation System (ECTS).

For the included public health programs, we conducted a more comprehensive data extraction of pre-defined information categories (in-depth PH program insights) from program websites and official documents e.g. regarding the general curriculum, specialization possibilities, accreditation forms or publishing requirements for doctoral programs. Relevant information for each program was extracted from program websites and supporting documentation (e.g., module handbooks). Information regarding a program’s accreditation was gathered from the German accreditation database [26]. Affiliations between program’s institution with pre-selected scientific associations were gathered from the respective associations’ websites. The selected associations included ASPHER, Deutsche Gesellschaft für Public Health (DGPH), Deutsche Gesellschaft für Epidemiologie (DGEpi), and Kooperationsverbund Hochschulen für Gesundheit (HOGE). In situations where information could not be found online, an email requesting the missing information was sent to the relevant study administrators/coordinators. Up to two emails distanced two weeks apart were sent, and if no reply was received then the information was recorded as missing. An overview of all extracted variables is given in Table 2.

**Table 2:** Variables of interest for data extraction

Variables of interest
*General variables extracted for all population health science programs (master’s & doctoral levels):*
▪ Program title ▪ Institution name ▪ Title awarded (M.Sc., M.A, Ph.D., Dr. PH, etc.) ▪ Institution type ▪ Institution location ▪ Form of study (part-time or / and full-time)^1^ ▪ ECTS ▪ Duration (regular / maximum)^2^
*In-depth variables extracted for public health programs (master’s & doctoral levels):*
▪ Curriculum (names of modules, categorization as core/electives) ▪ Distribution of ECTS between core and elective modules ▪ Inclusion of a mandatory internship/exchange (yes/no, duration, semester number) ▪ Competencies as listed on program’s website (qualitative) ▪ Employment possibilities (as listed on website) ▪ Admission requirements (Accepted bachelor’s degrees, classification as consecutive / non-consecutive, written entry exam (yes/no), admission interviews (yes/no), numerus clausus (yes/no, if yes: threshold)) ▪ Fees (per semester) ▪ University faculty to which program is associated (e.g., medicine, social sciences, etc.) ▪ Language of teaching (German / English / both / other) ▪ Teaching mode (in person, online or hybrid) ▪ Form and provider of accreditation and accreditation reviewer^3^ ▪ Membership to relevant scientific associations (e.g., ASHPER, DGPH, DGEpi, DGSMP, HOGE)
*In-depth variables extracted for public health doctoral programs:*
▪ Core curriculum (yes/no)
▪ Dissertation type (Monography, cumulative or individual choice)
▪ Requirements for scientific publications (required number of papers, authorship requirements (1st / 2nd / any position) and accepted types of paper (e.g., primary research only, additional acceptance of systematic reviews and / or study protocols), required journal rankings (e.g., impact factor, defined upper tier within the field) etc.)
*In-depth variables to be extracted if found (master’s & doctoral programs):*
▪ Number of admitted students per year ▪ Proportion of international students

*Abbrev*. *ASHPER* (Association of Schools of Public Health in the European Region), *DGEpi* (Deutsche Gesellschaft für Epidemiologie), *DGPH* (Deutsche Gesellschaft für Public Health), *DGSMP* (German Society for Social Medicine and Prevention), *Dr*. *PH* (Doctor of Public Health), *ECTS* (European Credit Transfer and Accumulation System), *HOGE* (Kooperationsverbund Hochschulen für Gesundheit e.V.), *M*.*A* (Master of Arts), *M*.*Sc*. (Master of Science), *Ph*.*D*. (Doctor of Philosophy)

^1^No program was identified falling into prior defined subcategories of “*in-service, or fully vocational” form of study*

^2^This information was originally planned to be extracted, but was often not available, and therefore excluded post-hoc from our mapping

^3^Extraction of this information was not pre-specified but added in the course of the data extraction process

Information on curricula of master’s and doctoral programs was retrieved from the respective program websites and categorized as core curriculum courses and mandatory elective courses (courses where students can choose from several options), not including voluntarily elective courses. Courses were grouped according to overarching topics in duplicate by GM and HS.

Several degree programs provided information in both English and German. Where available, the English information was initially used for screening and extraction. This was followed by a review of the German information to ensure that all relevant information was captured. In cases where information was available only in German, we translated the extracted information into English.

### Geographical mapping of programs

Geographic location of the institutions offering the included programs was extracted from the websites to identify clusters of academic education offers in the field of population health science and public health. A geographical map of all non-online programs was created using the open-source software R-Studio (R Version 4.3.2).

### Results syntheses

Where feasible, data was extracted in a quantitative manner to allow for summation, e.g. curriculum content, admission requirements and organization of supervision of doctoral dissertations. Absolute and relative frequencies (%) were presented for each category of these variables in tabular form. For variables where this was not possible (employment possibilities/work fields and acquired competencies), data was collected in a manner that allowed for a qualitative synthesis of results. This narrative synthesis was done by comparing relevant quotes extracted from programs’ websites and accompanying materials to identify common themes and categories.

## Results

### Search results

The search for master’s programs on the HEC was conducted on March 6, 2023 for the terms “Gesundheit” and “Health”, yielding *n* = 296 and *n* = 127 results, respectively, and on March 7, 2023 for the terms “epidemiology”, “Epidemiologie”, “Versorgung”, “Prävention” and “prevention”, with “Versorgung” yielding the most results (*n* = 56). After deduplication a total of *n* = 427 different master’s programs remained for screening. Three programs were added later during data extraction because two universities offered variations of their master’s programs to be achieved with either 120 or 60 ECTS and one university offered an online version in addition to their in person public health master’s program.

The search for doctoral programs was conducted on June 11, 2023 on the HEC, resulting in *n* = 23 identified programs, and on June 13, 2023 on Google, providing *n* = 96 results on the first ten pages. The search of the DAAD platform was conducted on June 26, 2023 and yielded *n* = 10 results. Additionally, six programs were identified through cross-references we found during the search and screening processes for master’s programs as well as during the extraction of information about master’s and doctoral programs from the respective web pages, resulting in a total of *n* = 68 doctoral programs after de-duplication.

**Fig. 2:**
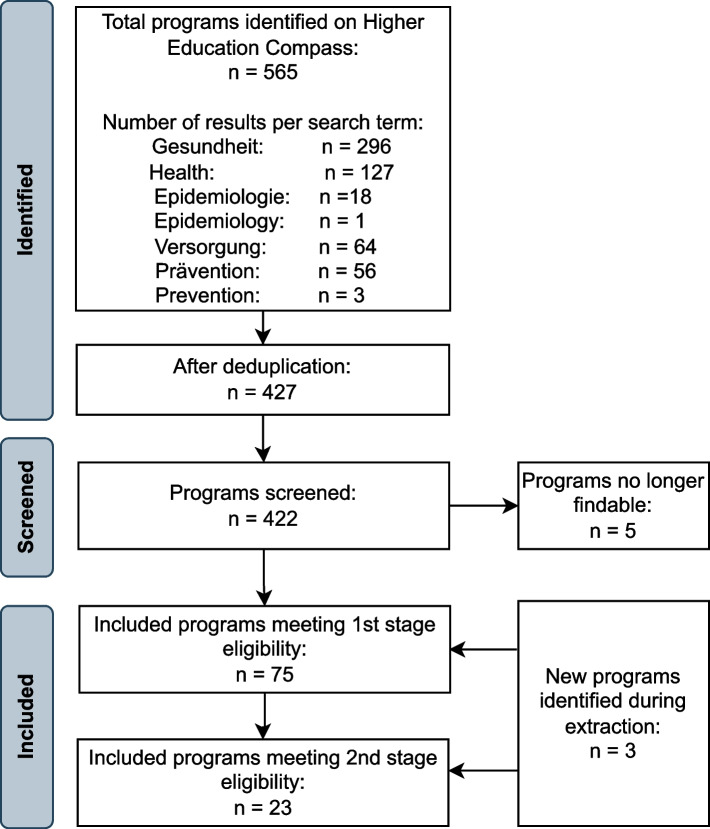
Identification of population health science and public health master’s programs

**Fig. 3:**
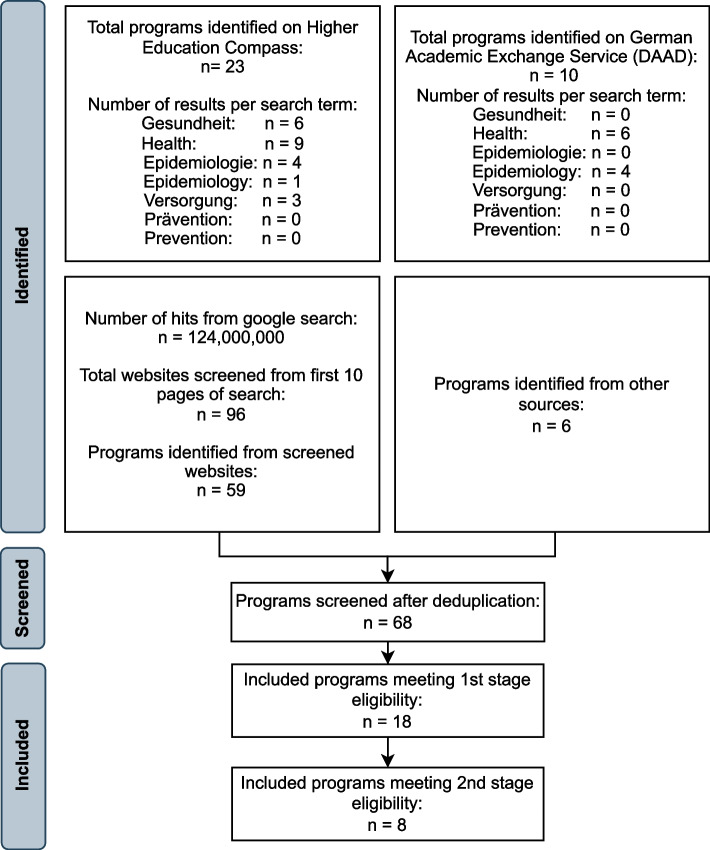
Identification of population health science and public health structured doctoral programs

After screening these records, a total of *n* = 75 master’s and *n* = 18 structured doctoral programs met the first-stage eligibility criteria and qualified as a population health science degree program for the overall PHS program mapping. Of these, a subgroup of *n* = 23 master’s and *n* = 8 doctoral programs met our second-stage eligibility criteria and were accordingly classified as public health programs for the in-depth program insights. The screening process is depicted in Figs. 2 and 3. An overview of included programs in this review and the website links from where the collected data was retrieved for master’s and doctoral programs can be found in Additional Files 2 and 3, respectively.

### Mapping the program landscape

#### Regional distribution of public health master’s and doctoral programs in Germany

**Fig. 4:**
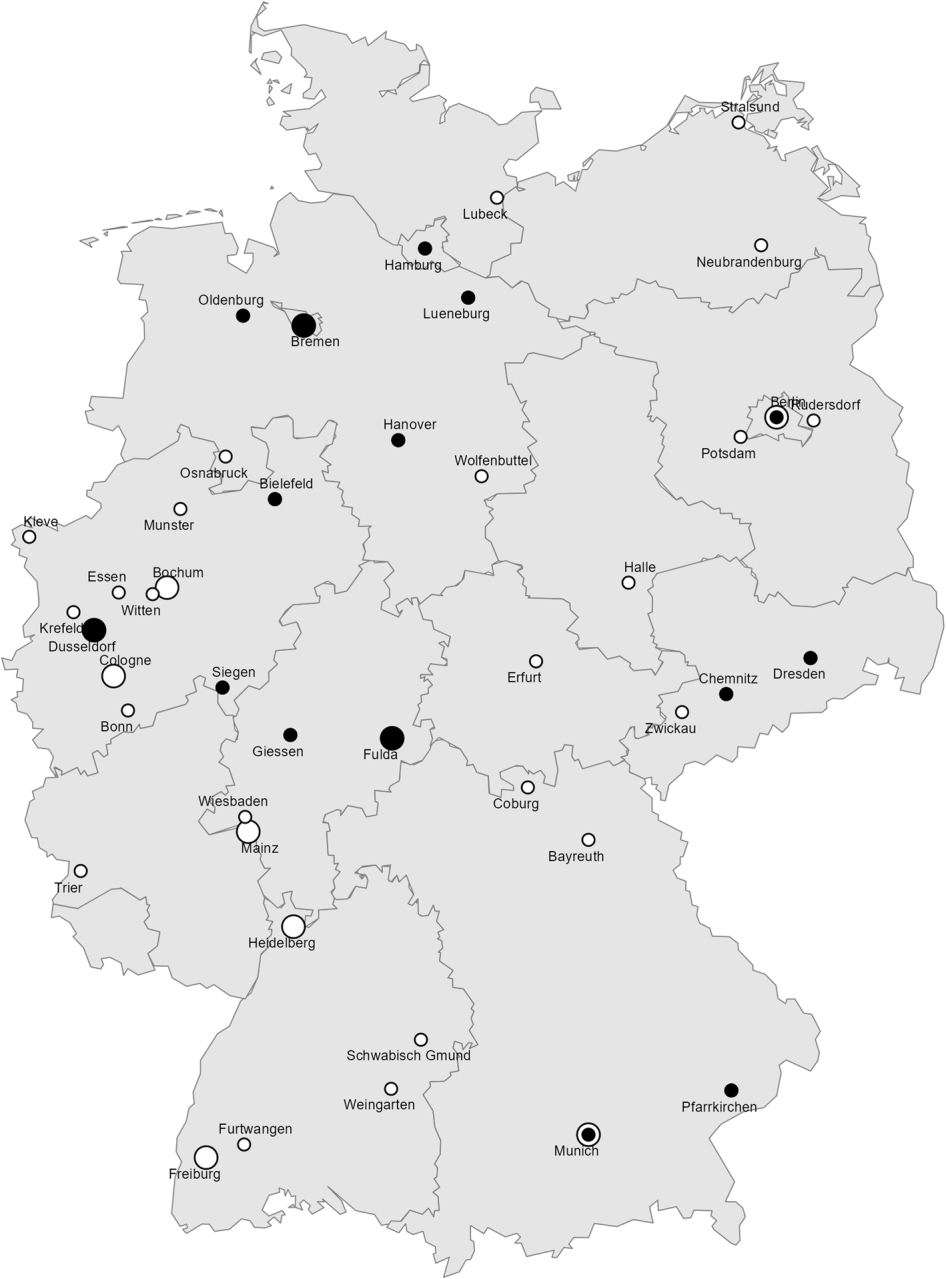
Locations of population health science and public health master’s programs. Circle size reflects the number of programs offered in a city. Black filled circle = public health master’s programs. White filled circle with black outline = population health science programs. Online programs are not included

**Fig. 5:**
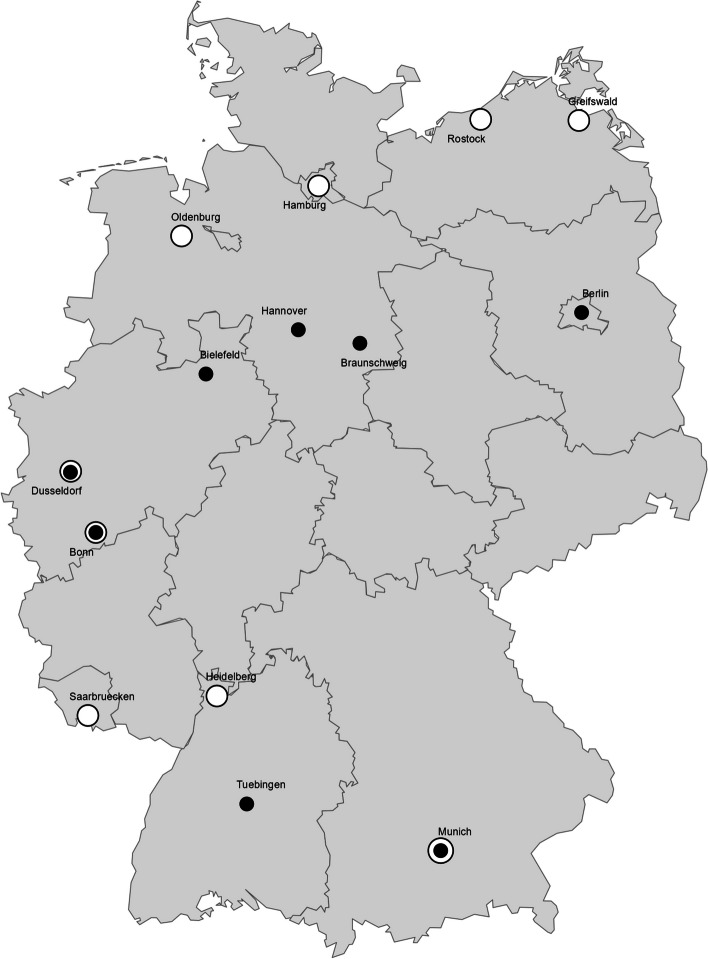
Locations of population health science and public health doctoral programs. Circle size reflects the number of programs offered in a city. Black filled circle = public health structured doctoral programs. White filled circle with black outline = population health science structured doctoral programs

Our regional mapping shows the geographical distribution of both master’s (Fig. 4) and structured doctoral programs (Fig. 5) in Germany. Four public health master’s programs which were offered online and one private university of applied science which offered a program in multiple cities are not included on the map. Master’s in health science programs were offered in all but two federal states (i.e. Saarland and Schleswig-Holstein), however public health master’s or structured doctoral programs were only offered in all but five federal states (Schleswig-Hostein, Mecklenburg-Vorpommern, Saxony-Anhalt, Thuringia and Rhineland-Palatinate). We found Munich to be the only city offering both population health science and public health programs on both master’s and doctoral level. Berlin, Bielefeld and Düsseldorf were found to offer both population health science and public health education, however, either only on doctoral or only on master’s level. The locations of all mapped master’s and doctoral programs can additionally be found in Additional Files 4 and 6 respectively.

#### Master’s programs – general mapping of population health science programs and details on public health programs in Germany

The summarized results of the general program mapping (see Table 3), which were extracted for all *n* = 75 population health science programs including the subset of *n* = 23 public health programs, showed that a majority of all programs (73.3%) awarded their graduates a Master of Science (M.Sc.) title, 10.7% a Master of Arts (M.A.) and 16% other titles (Master of Public Health (MPH), M.Sc. in International Health, M.Sc. in Epidemiology). Among the subgroup of public health programs, a comparatively smaller share of 60.9% programs awarded their graduates a M.Sc., and a larger share (30.4%) a MPH title. The complete program-level extracted structural data for all population health science master’s programs can be found in Additional File 4.

**Table 3:** Results for master’s programs

	**Population health science (including public health) ** (stage-1 eligible sample) 100% (*n *= 75)		**Public health** (stage-2 eligible sample) 100% (*n *= 23)^2^		**Non-public health population health science**^1^ (stage-2 ineligible sample) 100% (*n* = 52)	
*General information on master’s programs*						
**Title awarded**						
M.Sc.	73.3%	(55)	60.9%	(14)	78.9%	(41)
M.A	10.7%	(8)	8.7%	(2)	11.5%	(6)
Other (e.g. MPH, specialized M.Sc. degree)	16%	(12)	30.4%	(7)	9.6%	(5)
**Institution type**						
University	46.7%	(35)	39.1%	(9)	50%	(26)
University of Applied Sciences	49.3%	(37)	52.2%	(12)	48.1%	(25)
Cooperation between institutions	4%	(3)	8.7%	(2)	1.9%	(1)
**Form of study**						
Full-time	48%	(36)	43.5%	(10)	50%	(26)
Part-time	16%	(12)	17.4%	(4)	15.4%	(8)
Both forms possible	36%	(27)	39.1%	(9)	34.6%	(18)
**Total ECTS**						
120	68%	(51)	65.2%	(15)	69.2%	(36)
90	17.3%	(13)	21.7%	(5)	15.4%	(8)
60	16%	(12)	13.1%	(3)	11.5%	(6)
90 or 120	2.7%	(12)	0%		3.9%	(2)
**Regular duration (semesters)**^3^						
2	8%	(6)	4.4%	(1)	9.6%	(5)
3	13.3%	(10)	21.7%	(5)	9.6%	(5)
4	72%	(54)	65.2%	(15)	75%	(39)
5 or more	6.7%	(5)	8.7%	(2)	5.8%	(3)
			**Public health **(stage-2 eligible sample)100% (*n* = 23^2^)			
*Detailed information on public health master’s programs*						
**Workload master’s thesis (ECTS)**						
30			52.2%	(12)		
30, including colloquium			26.1%	(6)		
< 30			21.7%	(5)		
**Thesis colloquium**^4^						
Yes			39.1%	(9)		
No			60.9%	(14)		
**Mandatory internship **						
Yes			13%	(3)		
No			87%	(20)		
**Admission requirements**						
Bachelor degree with any background			100%	(23)		
Bachelor degree with a relevant background			65.2%	(15)		
Previous statistical and/or epidemiological experience			13%	(3)		
Aptitude test involving either written and/or oral components			34.8%	(8)		
Experience in a public health related position (minimum 1 year)			30.4%	(7)		
Internship (at least 3 month)			8.6%	(2)		
**Semester fees (€/ semester)**						
< 400			56.5%	(13) (Range: 72€-378,22€)		
> 400			43.5%	(10) (Range: 967,7€-2.910€)		
**Teaching language**						
German			73.9%	(17)		
English			8.7%	(2)		
German and English			17.4%	(4)		
**Mode of teaching**						
Online			17.4%	(4)		
In person			82.6%	(19)		
**Assigned faculty**						
Medical			17.4%	(4)		
Public health/ population health science			34.8%	(8)		
Social science			4.3%	(1)		
Other (e.g. nutrition, life science, not specified,…)			43.5%	(10)		
**Form of accreditation**^5^						
System accreditation			43.5%	(10)		
Program Accreditation			47.8%	(11)		
**Membership to Public Health association**^6^						
ASPHER			52.2% (12)	(12)		
DGPH			60.7%	(14)		
Other			8.7%	(2)		
Institutions with no indicated membership on the web-page			26.1%	(6)		
**Number of students enrolled per year**^7^						
≤ 30			58.3%	(7) (20-30 students/year)		
> 30			41.7%	(5) (45-100 students/year)		
**Average percentage of international students**^8^						
≤ 5%			44.4%	(4)		
5 -15%			44.4%	(4)		
90-95%			11.1%	(1)		

*Abbrev*. *ASHPER* (Association of Schools of Public Health in the European Region), *DGPH* (Deutsche Gesellschaft für Public Health), *ECTS* (European Credit Transfer and Accumulation System), *M*.*A* (Master of Arts), *M*.*Sc*. (Master of Science), *MPH* (Master of Public Health)

^1^Excluding programs that explicitly carried the term ”public health” in their title

^2^Programs of the same title with different course structures (e.g., amount of ECTS) were considered separate programs

^3^Programs that provided in months vs semesters were converted to the closest approx. semester duration e.g., 12 months = 2 semesters

^4^We defined a colloquium as consisting of a seminar or other course format of regular support alongside the thesis. We did not consider examination forms which are called ”colloquium” in some cases in this section

^5^Two programs were in process of gaining accreditation

^6^Some institutions have multiple memberships

^7^Information retrieved after personal email contact requesting this data, 52.2% response rate (*n* = 12)

^8^Information retrieved after personal email contact requesting this data, 39.1% response rate (*n* = 9), no programs with range of international students between 16 – 89%

Regarding the institution type, almost all master’s programs were provided by universities (46.7% overall) or universities of applied science (49.3% overall), with public health programs less frequently offered at universities (39.1%) and more often at universities of applied science (52.2%). Half of the population health science programs (48%) were designated as a full-time study, with 36% of programs allowing either full- or part-time study. The majority of population health science programs (68%) comprised 120 ECTS in total, with some programs requiring 90 ECTS or 60 ECTS. The regular duration of programs was four semesters for most population health science programs (72%), with a higher share of three-semester programs in the public health subsample (21.7% vs 13.3%). We also found some program durations of two or more than four semesters.

Further in-depth information on program structure and contents were extracted only for the subsample of *n* = 23 public health master’s programs (Table 3). The complete program-level extracted data from the public health master’s programs (in-depth program insights) can be found in Additional File 5.

#### Doctoral programs – overview of population health science programs and details on public health programs in Germany

The *n* = 18 doctoral population health science programs awarded 24 different titles, which we categorized in three groups (see Table 4): These included Doctor of Public Health (Dr. PH) (3 programs), and Doctor of Philosophy (Ph.D.) (14 programs). Furthermore, Doctoral Life Science titles, which are common for the traditional German doctoral system (e.g. Dr. rer. medic., Dr. rer. nat., Dr. rer. biol. hum.), were awarded by 7 programs. Within the subset of *n* = 8 public health doctoral programs, many programs awarded a choice between several titles. The full program-level list of extracted general data from the population health science doctoral programs (general PHS program mapping) can be found in Additional File 6.

**Table 4:** Results for doctoral programs

	**Population health science (including public health)** (stage-1 eligible sample) *n* = 18	**Public health** (stage-2 eligible sample) *n* = 8^1^	**Non-public health population health science** (stage-2 ineligible sample) *n* = 10
*General information on doctoral programs*			
**Title awarded**^2^			
Dr. PH	3	3	0
Ph.D.^3^	14	6	8
Life Science title (e.g. Dr. rer. medic.)	7	1	6
**Institution type**			
University	13	7	6
Cooperation between (applied) university and research institution	5	1	4
**Program duration**^4^			
2–3 years	2	1	1
3 years (without information on max. duration)	3	1	2
3 years (extension possible or no maximum duration)	4	1	3
3 years (max. 4)	4	2	2
3–5 years	3	1	2
**Form of Study**^5^			
Full-time	7	4	3
Part-time	0	0	0
Both forms possible	3	0	3
**Total ECTS**^6^			
180	5	3	2
Between 24–120	4	1	3
		**Public health** (stage-2 eligible sample) *n* = 8	
*Detailed information on public health doctoral programs*			
**Admission requirements**			
Population health science or public health relevant master’s degree		6	
Minimum six semesters completed		2	
Minimum 240 ECTS		1	
*State examinations in the subjects:*			
• medicine, dentistry or pharmacy		5	
• veterinary		3	
*Presentation of the intended dissertation project:*		5	
• written form		4	
• oral forms (presentation, interview)		3	
• written and oral form		2	
**Fees**^7^			
No fees		4	
No fees		1	
**Teaching language**			
German (dissertation in ENG or GER)		2	
English		4	
German and English		2	
**Mode of teaching**			
In person with some courses online		1	
Completely in person		7	
**Membership to Public Health association**^8^			
ASPHER		3	
DGPH		1	
Other		2	
Institutions with no indicated membership on the web-page		4	
**Assigned faculty**			
Medical		6	
Epidemiology/ population health science		2	
**Core curriculum**			
Yes		7	
No		1	
**Format of dissertation**			
Cumulative dissertation		1	
Self-selection (cumulative or monography)		7	
**Publication requirements for cumulative dissertation**^9^			
* Minimum number of first authorships required:*			
* 0*^10^		1	
* 1*		5	
* 2*		2	
* Minimum number of required accepted or published manuscripts:*			
1		3	
2		3	
3		1	
4		1	
* Journal prerequisites:*			
International and peer reviewed		6	
Peer-reviewed		1	
* International*		1	
**Minimum number of supervisors including mandatory co-supervisors or doctoral committee members**			
2		4	
3		3	
> 1 (exact number unclear)		1	

*Abbrev*. *Dr*. *PH* (Doctor of Public Health), *ECTS* (European Credit Transfer and Accumulation System), *Ph*.*D*. (Doctor of Philosophy), *Dr*. *rer*. *medic*. (Doctor rerum medicinalium, i. e. doctor of medical sciences)

^1^Programs found eligible either based on their title or program description

^2^Many programs awarded a choice between several titles

^3^Found with or without specification, such as Ph.D. in Medical Research – International Health, Ph.D. in Epidemiology & Public Health, Ph.D. in Public Health, or Ph.D. in Medical Sciences

^4^Two eligible public health doctoral programs provided no information on regular duration

^5^Eight programs did not provide information about form of study

^6^Nine programs either did not use ECTS credits or did not provide any information

^7^Three programs did not provide information about fees

^8^Some institutions have multiple memberships

^9^Information retrieved from online accessible sources provided by offered institution, may be incomplete

^10^One program does not state needed first authorships

The majority of doctoral population health science programs (13 out of 18 programs) and public health doctoral programs (7 out of 8 programs) were offered by universities, while remaining doctoral programs were offered through cooperations between universities/universities of applied sciences and research institutions. For the programs that used ECTS, the number of required credits varied from 24 to 180, with 30 ECTS most commonly dedicated to coursework. The complete program-level extracted data from the public health doctoral programs can be found in Additional File 7.

Admission requirements included a population health science or public health relevant master’s degree (6 programs). State examinations were accepted by five programs if they were in fields like medicine, dentistry or pharmacy, and by three programs in veterinary science. A presentation of the intended dissertation project was required by five programs. Equivalency of foreign degrees/diplomas was often left to the discretion of the dean or doctoral committee. No program included a mandatory internship or exchange abroad, but two institutions stated that external research stays were possible and supported. Accreditation information of public health doctoral programs was missing for six programs and two confirmed no existing accreditation via email.

Dissertation requirements varied between programs, including both monographic and cumulative formats or the option to choose between the two. The requirements were stated in the official examination or program regulations, but not all information was clearly accessible to public and therefore information may not be complete. The number of accepted or published papers, the prerequisites for authorship and journal type varied strongly. For example, one program required doctoral candidates to have lead authorship in one paper published in an international peer-reviewed journal or three papers in peer-reviewed journals, with a minimum of one lead authorship. Shared first authorships were allowed by several programs. Two programs requested the publication date of at least one publication not to be older than one year by the time of the doctoral examination procedure and six programs required publications in international and peer-reviewed journals.

Supervision of doctoral dissertations was organized in all eight programs through the formation of a committee consisting of a minimum of two (*n* = 4 programs) to three experienced researchers (*n* = 3 programs) to be responsible for each doctoral student. A common requirement for supervisors was to have teaching responsibilities either in professor positions or as private lecturers. Inclusion of external supervisors from other universities or institutions often was possible. Five of the programs specifically required at least one committee member to be from the medical faculty, although a specific faculty for the first supervisor was never predetermined by any program. The professional relationship allowed between the first and second supervisor was specified by four of the programs. This was often regulated with requirements for the second supervisor to belong to a different faculty or department.

### Curricula of programs

#### Courses offered within public health master’s programs

Table 5 provides an overview of core and mandatory elective courses of the included *n* = 23 public health master´s programs. The most frequently covered topics were epidemiology and public health (part of the core curriculum for *n* = 20 public health master’s programs). Further program core contents consisted of health systems research (*n* = 15 programs), prevention and health promotion (*n* = 12), health economics (*n* = 12) and health policy (*n* = 10). Methodologically, core curricula emphasized research methods (*n* = 16), statistics (*n* = 14), qualitative (*n* = 2) and quantitative methods (*n* = 1).

**Table 5:** Overview of the mandatory courses included in curricula of *n* = 23 public health master’s programs

Topics of the courses	Number of programs covering the topic in core curriculum	Number of programs covering the topic as mandatory elective	Number of programs covering the topic both in core curriculum and as mandatory elective	Total number of programs covering the topic either as core or mandatory elective courses
Epidemiology	20	6	5	21
Public health	20	4	4	20
Health systems (research)	15	6	3	18
Management	10	12	5	17
Research methods	16	1	1	16
Health economics	12	8	4	16
Statistics	14	7	5	16
Prevention and health promotion	12	6	4	14
Determinants of health	9	7	3	13
Sociology and social medicine	11	1	1	11
Global health	4	9	3	10
Environment and health	5	7	2	10
Health policy	10	3	3	10
Digital health/ digitalization	3	9	2	10
Other	3	8	1	10
Project work	8	0	0	8
Health psychology	7	1	1	7
Demography	4	4	1	7
Diversity	0	7	0	7
Health technology	0	6	0	6
Internship	3	3	0	6
Health communication	1	5	0	6
Quality and/or project management	5	1	0	6
Nutrition	1	5	0	6
Ethics	5	0	0	5
International health	4	1	0	5
Qualitative research methods	2	3	0	5
Work and health/occupational health	3	3	1	5
Health and society	2	2	0	4
Scientific writing	4	0	0	4
Health reporting	2	1	0	3
Mental health	1	2	0	3
Data science/management	0	3	0	3
Quantitative research methods	1	2	0	3
Population health science	1	2	0	3
Aging and health	1	3	1	3
Rehabilitation	1	3	1	3
Evidence-based medicine	1	2	0	3
Infectious diseases	0	3	0	3
Health case law	2	0	0	2
Health education	0	2	0	2
Medical basics	1	1	0	2
Health management	0	2	0	2
Behavioral science	1	0	0	1
Physical activity	1	0	0	1

#### Courses offered within public health doctoral programs

Table 6 provides an overview of core and mandatory elective courses of the *n* = 8 identified structured public health programs at doctoral level. Courses on good scientific practice were mandatory for six out of eight programs. Methodological research approaches and general health related background insights were provided for five of the eight programs, respectively. Courses supporting the individual research process such as colloquia, collaborative research seminars or journal clubs were offered in four programs, as were workshops, retreats, summer schools or conferences. One doctoral program offered no information on core curriculum, and one provided no information on mandatory electives.

**Table 6:** Overview of core and mandatory elective courses for *n* = 8 public health doctoral programs

Topics of the courses	Number of programs covering the topic in core curriculum^1^	Number of programs covering the topic as mandatory elective^2^	Number of programs covering the topic both in core curriculum and as mandatory elective	Total number of programs covering the topic either as core or mandatory elective courses
Good scientific practice	6	0	0	6
Methods courses	3	3	1	5
Coursework on broader research perspectives	2	4	1	5
Scientific events (conferences, workshops and summer schools)	0	4	0	4
Courses supporting individual dissertation process	2	2	0	4
Data science and statistics	2	1	0	3
Retreat	1	2	0	3
Presentation and scientific writing skills	0	2	0	2
Career planning	0	2	0	2
Epidemiological practice	1	0	0	1

^1^One program offered no information on core curriculum

^2^One program offered no information on mandatory elective courses

### Competencies and work fields

#### Competencies

The public health master’s programs intended to convey heterogenous sets of competencies. Some frequently mentioned learning objectives included deepening and expanding the knowledge and skills gained from previous academic education, such as the students’ scientific methodological skills required for the production and evaluation of scientific research. Further objectives included being capable of applying their knowledge and becoming proficient at identifying, implementing, and evaluating solutions to health problems on a population level. Also, interdisciplinary knowledge and skills required for translation of theory into practical real-world applications were stressed. Additionally, many programs aimed to convey an understanding of the determinants of health and illness, with several aiming to create an understanding of how health systems function at the regional, national, and international level.

All but one of the doctoral programs provided some form of information on the competencies to be acquired. They emphasized interdisciplinarity, highlighting the need for insights from different fields such as epidemiology, sociology, and economics. Key competences included conducting independent in-depth scientific research, developing methodological skills, and advanced professional qualifications for different career paths in public health and related fields. Teaching skills and transferable skills such as critical thinking were also highlighted. Overall, doctoral programs emphasized a holistic approach to deepening expertise in complex health issues and high-level methodological skills in the field of research and academia.

Competencies were seldom explicitly stated on the institution’s websites. KK and HS extracted this information when mentioned in the context of knowledge and skill gain or when listed as competencies. The program-level data extracted can be viewed in Additional File 8.

#### Work fields

All *n* = 23 master’s programs mentioned some form of work field for which they aimed to qualify their graduates. The programs generally suggested that by offering a broad education, they would produce well-educated professionals prepared for diverse roles, including leadership positions, but without clearly defined job titles or limitations to a specific workplace. The wide range of work fields mentioned by programs’ sites included academia, research, the Public Health Service, politics, health administration, organizations in health, care and social sectors, insurances, pharmaceutical companies, consulting agencies for private businesses as well as policy, occupational health and corporate health management. The importance of interdisciplinary work and holistic perspectives was stressed by programs, suggesting their graduates’ ability to work at the intersection of various sectors and institutions. Concrete tasks they suggested included teaching, advising, planning, assessing, evaluating, quality assurance, producing/ summarizing evidence, communicating, campaign/ project work. Further working fields mentioned comprised development aid, teaching in vocational institutions, medical wholesale and retail sector, health economics, the food industry, self-employment, engineering/planning offices, marketing/ controlling, human resource management, mental health and prevention or nutrition/ prevention of malnutrition, digitalization, city planning, and health-related urban development.

Five of the eight included doctoral programs in this review mentioned potential work fields, claiming to prepare individuals for versatile careers in academia, research, national health institutions, and international organizations, covering diverse fields such as public health, medical science, and applied medicine. They emphasized high quality in research, research methodology, and teaching. Furthermore, they stated that their graduates would be equipped for leadership and management roles in academic institutions, health policy, health and social services, as well as commercial healthcare facilities. Tasks mentioned varied from quality assurance to implementation and assessment of healthcare strategies.

The program-level data extracted regarding potential work fields can be viewed in Additional File 9.

## Discussion

With our systematic mapping, we identified 75 master’s and 18 structured doctoral programs with a population health science focus, amongst these 23 public health programs at master’s and 8 at doctoral level. This suggests an increase from 2014 where Hartmann and colleagues identified 17 master’s in the field of health science and public health, albeit with stricter inclusion criteria and more searched data bases [22].

Geographically, we identified Bielefeld, Düsseldorf, Berlin, and Munich as centers of academic knowledge and research in the field of public health in Germany. Only Munich seemed to offer both master’s and structured doctoral programs in both public health and population health science. This could suggest a lack of population health “educational hubs” in Germany, although the question arises as to whether bundled facilities are necessarily better than multiple distributed education facilities. Generally, public health specific structured doctoral programs remain scarce, and their curricula rather open in nature. Overall, the curricula show the broad, multi-, and interdisciplinary education provided across public health master’s programs in Germany, with some programs integrating with fields including nutrition, health economics, and environmental health. The results of our mapping align with the overview on public health education in South Asia by Anitha and colleagues [19] presenting heterogeneous curricula contents of public health programs that aim to educate their graduates with a broad set of skills and knowledge. As in programs in South Asia, most German public health programs emphasize public health, epidemiology and touch upon content areas of research on health systems, prevention and health promotion, health economics and health policies. We found no program offering courses on implementation science, which would ensure effectiveness of the implementation strategies tailored to the target population, implementing evidence-based practices, programs, or policies in healthcare [27]. This is surprising given the fact that the translation of research into policy and practice could be seen as one of the core tasks of the Public Health Service, one important potential future work field for public health graduates. Neither did we find public crisis/emergency training, despite the newly arising public health emergencies in Europe due to infectious diseases with pandemic potential, armed conflicts inducing national complex public health emergencies, and threats of extreme climate events [28]. We found that courses which explicitly teach methods to evaluate the effectiveness of (population-based) public health interventions seemed to be underrepresented in the curricula of health science programs. Also, courses on physical activity, behavioral science, health management, aging and health and mental health, although major public health topics, were rarely offered. Furthermore, we found that qualitative research methods were infrequently offered compared to quantitative research methods and statistics. This seems to reflect a common imbalance in public health research where quantitative research is more frequently represented in public health journals despite acknowledgement that qualitative methods offer equally valuable and complementary perspectives in public health research [29].

Overall, an open question that could not be answered from the curriculum mapping is how the curricula were compiled, i.e. the strategy along which courses were included. While there is obvious progress in defining and listing competencies and learning objectives for epidemiology [30] and biomedical and health informatics [31] at the country level in Germany, an overarching, consented national learning objective catalog for public health programs, is currently missing. Such consented lists of required competencies have previously been published by overarching public health associations e.g. the World Health Organization’s Essential Public Health Operations [32] or more regionally specific for Canada [33], the United States [34], and Europe [16]. Such a list might provide helpful orientation for newly developed programs or for providing public health accreditation for existing programs and to support continued alignment of program contents with evolving societal and stakeholder needs.

The large proportion of public health master’s programs offered costing more than 400€/semester (43.5%) indicates the high prevalence of potentially private or commercially driven institutions in the German public health education field. It was also interesting to observe that 13 public health master’s programs offered part-time study options and that 8 programs required only 60 or 90 ECTS for their degree compared to the more common 120 ECTS. These programs with a lower workload may indicate an interest or need for the ability of obtaining additional public health qualifications while already employed. Such flexible study options might be a means to facilitate the knowledge and skills acquisition required to bolster incorporation of professionals entering or transitioning into the Public Health Service workforce [9]. Overall, the future work fields suggested for both master’s and doctorate level public health programs seem to align well with the current actors and opportunities present in the German public health environment [35]. Preparation of students for the workforce however, often requires more than the acquisition of knowledge and certain competencies. Employers in the German public health workforce are also looking for practical experiences in addition to education, which can be achieved for example through involvement in extracurricular activities, volunteering, and internships [36]. Beyond providing practical experience, such opportunities also can enable students to build valuable networks to access and learn about relevant employment opportunities [36]. Interestingly, we found that an internship was only offered by three public health master’s programs as a core curriculum component and, additionally, found within the mandatory electives for three other programs.

Structured doctoral programs in the field of public health were mainly offered by medical faculties, which is, on the one hand, not surprising given the evolution of public health as its own discipline from the medical field and the close required collaboration between the two. On the other hand, it could be argued that public health and medicine, might be equally well placed under a common umbrella structure such as health-focused schools or faculties, together with other relevant disciplines such as sociology or political sciences, which could foster interdisciplinary perspectives, exchange and collaborations.

In contrast to master’s programs, the focus of courses accompanying the doctoral programs lies more on supporting the individual research process. Possibly because doctoral topics are very individual, the curriculum was often methodologically oriented. We also found much variability in the process of obtaining a public health doctoral degree in Germany. This pertained for example to the titles awarded and the lack of uniform requirements regarding the number of authorship positions on publications for cumulative dissertations as well as on journal prerequisites, such as international and peer reviewed journals. Furthermore, supervision is organized differently in each public health doctoral program and the regulations and curricula vary strongly. This may make comparability of degrees difficult not only for external stakeholders, but even within the academic community*.*

### Strengths and limitations

To the best our knowledge, this is the first structured and comprehensive mapping of population health science and public health master’s and doctoral level academic programs available in Germany. We followed a pre-specified and published protocol, and where applicable, systematic review best practices such as duplication of the selection and data extraction process to ensure transparency, replicability, and comprehensiveness of our approach. In addition, this will allow for easier updates of this work by us or interested others, as the public health academic landscape continues developing.

There are some limitations of this study. Firstly, it should be considered that our mapping only provides a snapshot in time, reflecting the population health science and public health programs offered at the time of the search. Therefore, dynamics from new and existing programs are under development were not possible to capture. Furthermore, as HEC was our only source for capturing master’s programs, any relevant programs not listed on HEC would not have been included in our mapping.

Second, we only assessed the included public health education programs through the information provided on the respective program websites. These sources may provide limited or incomplete information on some topics of interest, such as competencies, potential fields of work and existing networks with academic and non-academic stakeholders. To understand the directions and future development of public health education programs in Germany, the experiences and perspectives of master’s and doctoral public health program heads, managers and coordinators in creating and developing public health programs should be additionally explored. Such a qualitative endeavor could be a valuable complement to our structured mapping approach by compiling best practice examples in the management and scientific coordination of public health master’s and doctoral programs as well as the main challenges encountered on this way. We hope to both build upon our findings and close some of these remaining information gaps in a further planned study, where we will use semi-structured interviews with program coordinators to add insights on such less accessible but nonetheless relevant aspects of study program maintenance and development.

Third, for some programs the information we were able to extract from publicly available sources remained incomplete. We contacted all programs’ study coordinators at least twice to minimize data gaps before marking information as not available. Still, this may have led to an unbalanced reporting of results for some variables, such as number of enrolled students and ratio of international students. Likewise, information about some doctoral programs, e.g. on publication requirements or the exact components of the curriculum may only be accessible in internal documents.

Finally, regarding doctoral programs, it is a limitation that only structured programs were included in our mapping. However, we did not consider it feasible to systematically review individually set-up and supervised doctoral programs. Our search of doctoral programs was also less structured (including a Google search) than the one we conducted for master’s programs, even though we explicitly shared our search strategy and attempted to make all choices transparent. The intention of this multifaceted search approach, however, was to maximize the likelihood of identifying all relevant public health doctoral programs.

### Practical implications

Building an understanding of the current state of public health education in Germany is a pre-requisite to enable better interconnection, joint development of shared teaching contents, objectives, and target competencies across programs, educational institutions, and educators. Furthermore, current and prospective students may use the information compiled in this study to make comparisons between programs and make informed decisions depending on individual preferences for their own educational and occupational paths. Cooperations between different universities offering public health programs could allow synergies to enable the provision of broader public health relevant contents. As already suggested by Hartmann and colleagues [22], we agree that the creation of an overarching public health core curriculum could bundle the knowledge transferred to the future public health work force to better tackle national challenges. This would also allow continuous development alongside international standards. Doctoral degrees in the field of public health should be better defined in accessible program descriptions and their requirements should be unified.

Furthermore, this comprehensive overview of public health education in Germany could play a role in fostering improved interaction between the academic and non-academic stakeholders of the public health sector in Germany and internationally. Public health institutions and other future employers can gain insights into the teaching contents and competencies of their future workforce which might support integration of graduates into the job market. In turn, allowing for establishment of better connections between theory and practice, and improved exchange of contents and best practice solutions might in the long-term support the development of a public health workforce that has all the competencies required to confidently tackle the public health and health systems challenges ahead. As discussed earlier, there is room for debate on whether the public health sector would benefit more from consolidating expertise in specialized hubs or distributing it across various academic institutions. Additionally, the inquiry into whether public health programs are best situated within a medical faculty, or if an alternative structural approach is more suitable, is also a relevant consideration in this context.

Our results may also be beneficial for other countries experiencing a need to improve their public health sector by developing public health programs, therefore our example could encourage similar mapping projects to be undertaken. One example is Ukraine, where (even though massively impeded by the ongoing war [37] the past decades have seen multiple reforms of the public health sector [38], particularly regarding financing mechanisms, digitalization of health care, primary care delivery, the creation of the central and regional centers of disease control and prevention on the basis of existing laboratory centers, as well as active development of the private sector of medical services. One powerful step meant to ensure the effectiveness of these reforms was the initiation of educational programs in the specialty of public health at bachelor’s, master’s, and doctoral levels to actively support developing future public health specialists [39].

While historically the understanding of public health and hygiene was also shaped by many influential scientists from Germany, such as Robert Koch and Rudolf Virchow, the discipline was largely discontinued in Germany after World War II due to perversion and abuse of population health terms and concepts during the Third Reich [40]. Thereafter, advancements in public health were largely driven by Anglo-Saxon countries. Only recently there is a renewed appreciation of modern public health concepts beyond clinically oriented disciplines, and public health research and education has gained more traction in Germany. Countries at an early stage of public health higher education program development may profit from this overview and may learn from Germany’s relatively recent development of a modern public health higher education system.

## Conclusion

This study provides a nuanced, comprehensive and up-to-date overview of public health education in Germany, highlighting its interdisciplinary nature. The findings, while acknowledging limitations, hold significant value in informing educational decisions for institutions, educators, and students. Programs in public health are offered with great diversity in their curriculum, allowing a wide range of specializations and therefore educating an interdisciplinary work force in Germany. A comprehensive core curriculum for public health programs that defines the overarching core competencies and allows for specialization could further benefit the public health sector in Germany. This would also allow to align public health education with overarching societal health objectives, ensuring coherence and relevance to the specific health goals of each country. We hope that the results of our mapping can support and improve mutual learning, exchange and collaboration between institutions, and harmonization of program structures and curricula through increased transparency and potentially collection of best practice examples, and that, ultimately, this research supports development of competent professionals capable of meeting the challenges and complexities of evolving, dynamic population health needs within and beyond health systems.

### Supplementary Information

Additional file 1. (Short overview of higher education organization in Germany)

Additional file 2. (Institution and title of master’s programs, including website links)

Additional file 3. (Institution and title of doctoral programs, including website links)

Additional file 4. (Extracted general data for population health science master’s programs)

Additional file 5. (Extracted detailed data for public health master’s programs)

Additional file 6. (Extracted general data for population health science doctoral programs)

Additional file 7. (Extracted detailed data for public health doctoral programs)

Additional file 8. (Extracted data on competencies aimed at by public health master’s and doctoral programs)

Additional file 9. (Extracted data on work fields suggested by public health master’s and doctoral programs)

## Data Availability

All data generated or analyzed during this study are included in this published article and its online supplementary materials. All links to the programs’ websites are provided in the supplementary information files. Additional information that needed to be requested via email is marked accordingly in respective result table.

## Abbreviations

SARS-CoV-2: Severe acute respiratory syndrome coronavirus type 2
APHEA: Agency for Accreditation in Public Health Education
ASPHER: Association of Schools of Public Health in the European Region
PHS: Population health science
PH: Public health
PRISMA: Preferred Reporting Items for Systematic Reviews and Meta-Analyses
HEC: Higher Education Compass
DAAD: German Academic Exchange Service
MPH: Master’s in Public Health
ECTS: European Credit Transfer and Accumulation System
DGPH: Deutsche Gesellschaft für Public Health (German Public Health Association)
DGEpi: Deutsche Gesellschaft für Epidemiologie (German Society for Epidemiology)
HOGE: Kooperationsverbund Hochschulen für Gesundheit e.V. (Registered Cooperation Association for Universities of Health)
EUGLOH: European University Alliance for Global Health
DGSMP: German Society for Social Medicine and Prevention
M.Sc.: Master of Science
M.A.: Master of Arts
Ph.D.: Doctor of Philosophy
Dr. PH: Doctor of Public Health
