# Supplementary material for: A systematic mapping of public health master’s and structured doctoral programs in Germany
Source: BMC Med Educ. 2024 Aug 13;24:872. doi: 10.1186/s12909-024-05855-8 (PMC11323405; doi:10.1186/s12909-024-05855-8)
Supplement: Supplementary file 1 — Additional file 1. (Short overview of higher education organization in Germany) [file 12909_2024_5855_MOESM1_ESM.pdf]

Background information on the higher education system in Germany

The German higher education system is complex with a range of legislations and regulations present simultaneously at both the national and federal level (1). Master's programs in Germany are provided by a variety of institutions including universities, technical universities, and universities of applied science (“*Fachhochschulen”)* (1). Universities of applied science were previously responsible for vocational training of specific professional figures, and the diplomas they issued were not equivalent to those obtained at the other two types of universities. This has changed with the Bologna process, such that all master degrees obtained from all above mentioned higher education institutions in Germany are now objectively considered equal (1), reflecting the International Standard Classification of Education category 7 (2).

The Bologna process implied new accreditation requirements for master programs, with the respective regulations laid out in the Interstate Study Accreditation Treaty enacted in 2018 (3). This has increased equality and formal comparability of all public health master's programs (4) and entitles holders of master's degrees from all three educational institution types to pursue a doctoral degree (1). Despite these reforms' successes in establishing formal equivalence between university degrees, this equivalence has not extended to Public Health core curricula and core competencies which can differ substantially not only across university types, but even between individual public health programs offered by different universities of the same type (4).

Like master's degrees, also doctorate degrees including Ph.D.'s are obtainable in Germany at universities and equivalent institutions of higher education (1). In addition to the Ph.D. titles conferred by fully structured Ph.D. programs, a variety of other doctorate titles are commonly awarded in Germany. These specifically indicate the discipline for which they were awarded (e.g., Dr. Ph. for a Public Health focus, Dr. Biol. Hum. for a human biology focus, Dr. rer nat. for a natural science focus, or Dr. phil. for a social science focus). The exact processes through which these degrees can be obtained vary between institutions, with the majority of doctoral

degrees awarded through individual supervision without a formal thematic curriculum (sometimes under the umbrella structure of graduate schools), although fully structured Ph.D. programs are increasingly developed and are gaining importance (1). Within the scope of this study, we only included doctoral programs that are awarding doctoral degree through structured thematic programs.

References:

1. EURYDICE. The Education System in the Federal Republic of Germany 2018/2019 [Internet]. Eckhardt T, editor. Secretariat of the Standing Conference of the Ministers of Education and Cultural Affairs of the Länder in the Federal Republic of Germany; 2019. Available from: https://www.kmk.org/fileadmin/Dateien/pdf/Eurydice/Bildungswesen-engl-pdfs/dossier_en_ebook.pdf

2. The World Bank. Education Statistics | International Standard Classification of Education (ISCED) [Internet]. [cited 2023 Jan 30]. Available from: https://datatopics.worldbank.org/education/wRsc/classification

3. Kultusminister Konferenz. Interstate Study Accreditation Treaty [Internet]. Jan 1, 2018. Available from: https://www.akkreditierungsrat.de/sites/default/files/downloads/2021/161208_Studienakkreditierungsstaatsvertrag_mit%20Begruendung_Englisch.pdf

4. Deutsche Gesellschaft für Public Health e.V. Aktuelle Situation [Internet]. [cited 2022 Nov 7]. Available from: https://www.dgph.info/info-ueberblick/studiengaenge/phs-situation/
