# Supplementary material for: A systematic mapping of public health master’s and structured doctoral programs in Germany
Source: BMC Med Educ. 2024 Aug 13;24:872. doi: 10.1186/s12909-024-05855-8 (PMC11323405; doi:10.1186/s12909-024-05855-8)
Supplement: Supplementary file 2 — Additional file 2. (Institution and title of master’s programs, including website links) [file 12909_2024_5855_MOESM2_ESM.pdf]

Additional File 2 – Overview of mapped population health science and public health master's programs*

Institution:	Program title:	Program link(s):	Eligibility stage:
University Bielefeld	Public Health	http://ekvv.uni-bielefeld.de/sinfo/publ/master-as/pubhealth?&lang=DE	second
Charité AND Technical University of Berlin AND Alice Salomon Hochschule Berlin	Public Health	https://bsph.charite.de/studienangebot/public_health/ ; https://www.ash-berlin.eu/studium/studiengaenge/master-public-health/profil/	second
Technical University of Dresden	Gesundheitswissenschaften - Public Health	https://tu-dresden.de/med/mf/studium/ph	second
APOLLON Hochschule der Gesundheitswirtschaft	Public Health - Prevention and Mental Health	https://www.apollon-hochschule.de/fernstudium/master-public-health/	second
Heinrich-Heine-Universität Düsseldorf	Public Health	https://www.hhu.de/studium/studienangebot/studiengang-informationen?tx_igstudiengaenge_txigstudiengaengepi2%5Baction%5D=list&tx_igstudiengaenge_txigstudiengaengepi2%5Bcontroller%5D=DoSvDetail&tx_igstudiengaenge_txigstudiengaengepi2%5Bstudiengang%5D=322&cHash=8be8943a24450b76b5e07d13071acc80 ; https://www.public-health.hhu.de/start.html	second
Heinrich-Heine-Universität Düsseldorf AND Akademie für Öffentliches Gesundheitswesen	Public Health	https://www.hhu.de/studium/studienangebot/studiengang-informationen?tx_igstudiengaenge_txigstudiengaengepi2%5Baction%5D=list&tx_igstudiengaenge_txigstudiengaengepi2%5Bcontroller%5D=DoSvDetail&tx_igstudiengaenge_txigstudiengaengepi2%5Bstudiengang%5D=322&cHash=8be8943a24450b76b5e07d13071acc80 ; https://www.public-health.hhu.de/start.html ; https://www.akademie-oegw.de/aus-und-weiterbildung/master-of-science-in-public-health	second
Jade Hochschule - Wilhelmshaven/Oldenburg/Elsfleth	Public Health	https://www.jade-hs.de/unsere-hochschule/fachbereiche/bgg/tgm/studium/ph/	second
FOM Hochschule für Oekonomie & Management - University of Applied Sciences	Public Health	https://www.fom.de/de/hochschulbereiche/gesundheit-und-soziales/public-health-ma.html	second
Hochschule Fulda - University of Applied Sciences	Public Health	https://www.hs-fulda.de/gesundheitswissenschaften/studium/masterstudiengaenge/public-health-msc; https://www.hs-fulda.de/orientieren/meine-bewerbung/studiengaenge/details/studiengang/public-health-msc-1/show	second
Hochschule Fulda - University of Applied Sciences	Public Health Nutrition	https://www.hs-fulda.de/oecotrophologie/studium/master-studiengaenge/public-health-nutrition-msc	second
Universität Bremen	Public Health - Gesundheitsförderung und Prävention	https://www.uni-bremen.de/studium/orientieren-bewerben/studienangebot/dbs/study/102?cHash=a046b40e81d090b194c704a3b62afeb3 ; https://www.uni-bremen.de/index.php?id=5109	second
Universität Bremen	Public Health - Gesundheitsversorgung, -ökonomie und -management	https://www.uni-bremen.de/studium/orientieren-bewerben/studienangebot/dbs/study/103?cHash=de8fac9ab979c02089ccbe5e2ce74dee	second

*In the order they were found during the search.

Technische Universität Chemnitz	Public Health mit Schwerpunkt Prävention und Evaluation	https://www.tu-chemnitz.de/studierendenservice/zsb/studiengaenge/en/#88_B44	second
IU Internationale Hochschule	Public Health (120)	https://www.iu-fernstudium.de/master/public-health/120-de/ ; https://www.iu-fernstudium.de/master/public-health/?clickref=1100lwFZv8i3&utm_source=private-network&utm_medium=koop&utm_campaign=partner1011l39091&utm_content=	second
IU Internationale Hochschule	Public Health (60)	https://www.iu-fernstudium.de/master/public-health/60-de/	second
Technische Hochschule Mittelhessen - THM	Public Health	https://www.thm.de/site/studium/unsere-studienangebote/public-health-master-msc-ges-giessen.html	second
Hochschule für Angewandte Wissenschaften Hamburg	Public Health	https://www.haw-hamburg.de/en/study/degree-courses-a-z/study-courses-in-detail/course/courses/show/public-health/Studieninteressierte/	second
Ludwig-Maximilians-Universität München	Public Health	https://www.lmu.de/de/studium/studienangebot/alle-studienfaecher-und-studiengaenge/public-health-master-hauptfach-4550.html	second
Medizinische Hochschule Hannover (MHH)	Bevölkerungsmedizin und Gesundheitswesen (Public Health)	https://www.mhh.de/studium-public-health	second
Universität Siegen	Digital Public Health	https://www.uni-siegen.de/zsb/studienangebot/master/dph.html	second
Technische Hochschule Deggendorf	Global Public Health	https://www.th-deg.de/gph-m-en	second
Leuphana Universität Lüneburg	Prävention und Gesundheitsförderung	https://www.leuphana.de/professional-school/berufsbegleitende-master-mba/public-health-studium.html	second
APOLLON Hochschule der Gesundheitswirtschaft	Public health - Umwelt & Gesundheit	https://www.apollon-hochschule.de/fernstudium/master/master-public-health-umwelt-gesundheit/).	second
Charité - Universitätsmedizin Berlin, Technische Universität Berlin, Alice Salomon Hochschule (Berlin School of Public Health)	Epidemiologie	https://bsph.charite.de/studienangebot/epidemiologie/msc_epidemiology_am_institut_fuer_public_health/	first
Universität Bremen	Epidemiologie	https://www.uni-bremen.de/studium/orientieren-bewerben/studienangebot/dbs/study/98?cHash=6d6342e810b9f736553a45c8b82d47e2	first
Ludwig-Maximilians-Universität München	Epidemiologie	https://www.en.ibe.med.uni-muenchen.de/academics/epidemiology/index.html	first
Johannes Gutenberg-Universität Mainz	Epidemiologie (a - weiterbilend)	https://www.unimedizin-mainz.de/imbei/imbei/mse/postgradualer-master.html	first
Johannes Gutenberg-Universität Mainz	Epidemiologie (b - Konsekutiv)	https://www.unimedizin-mainz.de/imbei/imbei/mse/konsekutiver-master.html	first
Charité - Universitätsmedizin Berlin	Applied Epidemiology	https://bsph.charite.de/studienangebot/epidemiologie/msc_applied_epidemiology_am_robert_koch_institut/	first
Hochschule Furtwangen - Informatik, Technik, Wirtschaft, Medien, Gesundheit	Angewandte Gesundheitsförderung	https://www.hs-furtwangen.de/fakultaeten/gesundheit-sicherheit-gesellschaft/	first
Hochschule Ravensburg-Weingarten	Angewandte Gesundheitswissenschaft	https://www.rwu.de/studieren/studiengaenge/angewandte-gesundheits-wissenschaft	first

*In the order they were found during the search.

Westsächsische Hochschule Zwickau	Angewandte Gesundheitswissenschaften	https://www.fh-zwickau.de/studium/studieninteressenten/studienangebot/angewandte-gesundheitswissenschaften-master-berufsbegleitend/	first
Hochschule für Gesundheit - University of Applied Sciences	Angewandte Gesundheitswissenschaften	https://www.hs-gesundheit.de/studium/unser-studienangebot/angewandte-gesundheitswissenschaften/uebersicht-angewandte-gesundheitswissenschaften	first
Fachhochschule der Diakonie - Diaconia - University of Applied Sciences	Community Mental Health	https://www.fh-diakonie.de/.cms/studiengang/community_mental_health_master_studium/452	first
FH Münster University of Applied Sciences	Ernährung und Gesundheit	https://www.fh-muenster.de/studium/studiengaenge/index.php?studId=136	first
Hochschule für Gesundheit - University of Applied Sciences	Evidence-based Health Care	https://www.hs-gesundheit.de/studium/unser-studienangebot/evidence-based-health-care/uebersicht	first
Martin-Luther-Universität Halle-Wittenberg	Gesundheits- und Pflegewissenschaften	https://studienangebot.uni-halle.de/gesundheits-und-pflegewissenschaften-master-120	first
Universität zu Lübeck	Gesundheits- und Versorgungswissenschaften	https://www.uni-luebeck.de/studium/studiengaenge/gesundheits-und-versorgungswissenschaften.html	first
Europäische Fachhochschule Rhein/Erft, european university of applied sciences	Gesundheitsforschung und Therapiewissenschaften	https://www.eufh.de/master/gesundheitsforschung-therapiewissenschaften	first
Hochschule für angewandte Wissenschaften Coburg	Gesundheitsförderung	https://www.hs-coburg.de/studium/master/soziales-gesundheit/gesundheitsfoerderung.html	first
Pädagogische Hochschule Schwäbisch Gmünd	Gesundheitsförderung und Prävention	https://gfp.ph-gmuend.de/	first
Universität Erfurt	Gesundheitskommunikation	https://www.uni-erfurt.de/index.php?id=131	first
Pädagogische Hochschule Freiburg	Gesundheitspädagogik	https://www.ph-freiburg.de/studium/masterstudiengaenge/gesundheitspaedagogik.html	first
Hochschule Neubrandenburg - University of Applied Sciences	Gesundheitswissenschaften	https://www.hs-nb.de/studiengaenge/master/gesundheitswissenschaften-msc/	first
Westsächsische Hochschule Zwickau	Gesundheitswissenschaften	https://www.fh-zwickau.de/studium/studieninteressenten/studienangebot/gesundheitswissenschaften-master/	first
Hochschule Rhein-Waal - University of Applied Sciences	Gesundheitswissenschaften und -management	https://www.hochschule-rhein-waal.de/de/fakultaeten/life-sciences/studienangebot/gesundheitswissenschaften-und-management-m-sc	first
Universität Bayreuth	Gesundheitsökonomie	https://www.goe.uni-bayreuth.de/de/master/index.html	first
Universität zu Köln	Gesundheitsökonomie	https://wiso.uni-koeln.de/de/studium/master/master-gesundheitsoekonomie/	first
Hochschule Stralsund	Gesundheitsökonomie	https://www.hochschule-stralsund.de/goek/	first
APOLLON Hochschule der Gesundheitswirtschaft	Gesundheitsökonomie	https://www.apollon-hochschule.de/fernstudium/master-gesundheitsoekonomie/	first
Universität Duisburg-Essen	Gesundheitsökonomik	https://www.uni-due.de/studienangebote/studiengang.php?id=19	first
Universität Bielefeld	Health Administration	https://mha.uni-bielefeld.de/	first

*In the order they were found during the search.

Hochschule Niederrhein	Health Care – Gesundheitswissenschaften	https://www.hs-niederrhein.de/studienangebot/studiengang/m-sc-health-care/	first
Hochschule Osnabrück	HELPP - Versorgungsforschung und -gestaltung	https://www.hs-osnabrueck.de/studium/studienangebot/master/helpp-versorgungsforschung-und-gestaltung-msc/	first
Universität Potsdam	Integrative Sport-, Bewegungs- und Gesundheitswissenschaft	https://www.uni-potsdam.de/de/studium/studienangebot/masterstudium/master-a-z/isbgw.html	first
Hochschule Braunschweig/Wolfenbüttel, Ostfalia Hochschule für angewandte Wissenschaften	Integriertes Versorgungsmanagement im Gesundheitswesen	https://www.ostfalia.de/cms/de/studienberatung/studienangebot/integriertes-versorgungsmanagement-im-gesundheitswesen/	first
Albert-Ludwigs-Universität Freiburg im Breisgau AND Hochschule Furtwangen	Interdisziplinäre Gesundheitsförderung	https://www.studium.uni-freiburg.de/de/studienangebot/studienfaecher/info/696; https://www.igf-studium.de/angebote/master-of-science/	first
Charité Universitätsmedizin Berlin	International Health	https://internationalhealth.charite.de/en/study_programmes/master_of_science_in_international_health/	first
Universität Trier	Interprofessionelle Gesundheitsversorgung	https://www.uni-trier.de/studium/studienangebot/studienfaecher/studiengang?sgaid=246&cHash=5489286e2265453cea24386dfb8465bc	first
Carl von Ossietzky Universität Oldenburg	Versorgungsforschung	https://uol.de/studiengang/versorgungsforschung-master-620 120	first
Ruprecht-Karls-Universität Heidelberg	Versorgungsforschung und Implementierungswissenschaft im Gesundheitswesen	https://www.uni-heidelberg.de/de/studium/alle-studienfaecher/versorgungsforschung-und-implementierungswissenschaft-im-gesundheitswesen	first
Universität zu Köln	Versorgungswissenschaft	https://studienorientierung.uni-koeln.de/studienangebot/index_ger.html?app=true&id=368	first
Universität Bremen	Community Health Care and Nursing: Versorgungsforschung und Versorgungsplanung	https://www.uni-bremen.de/studium/orientieren-bewerben/studienangebot/dbs/study/97?cHash=8c14eeac616ecf69f547a14224561823	first
Private Universität Witten/Herdecke gGmbH	Community Health Nursing	https://www.uni-wh.de/studium/studiengaenge/community-health-nursing-m-sc/	first
Europäische Fachhochschule Rhein/Erft, european university of applied sciences	Global Health	https://www.eufh.de/master/global-health	first
Rheinische Friedrich-Wilhelms-Universität Bonn	Global Health Risk Management & Hygiene Policies	https://www.uni-bonn.de/de/studium/studienangebot/studiengaenge-a-z/global-health-risk-management-hygiene-policies-wm	first
Albert-Ludwigs-Universität Freiburg im Breisgau	Global Urban Health	https://mscglobalurbanhealth.uni-freiburg.de/	first
Universität zu Köln	Health Economics	https://wiso.uni-koeln.de/en/studies/master/master-health-economics/	first
Technische Universität München	Health Science - Prevention and Health Promotion	https://www.sg.tum.de/sg/studium/studierende/studiengaenge/masterstudiengaenge-msc/msc-health-science/	first
Hochschule für Angewandte Wissenschaften Hamburg	Health Sciences	https://www.haw-hamburg.de/en/study/degree-courses-a-z/study-courses-in-detail/course/courses/show/health-sciences/Studieninteressierte/	first

*In the order they were found during the search.

Ruprecht-Karls-Universität Heidelberg	International Health	https://www.uni-heidelberg.de/en/study/all-subjects/international-health/international-health-master-continuing-education	first
Ludwig-Maximilians-Universität München	International Health	https://www.cih.lmu.de/education/study-programs/master-programs/msc-int-health	first
Hochschule Fresenius	International Health Economics & Pharmacoeconomics	https://www.hs-fresenius.com/study-programs/international-health-economics-master/?crmid=bBcWvNcDaBaCbg	first
Katholische Stiftungshochschule für angewandte Wissenschaften München - Hochschule der Kirchlichen Stiftung des öffentlichen Rechts "Katholische Bildungsstätten für Sozialberufe in Bayern"	Angewandte Versorgungsforschung	https://www.ksh-muenchen.de/hochschule/campus-muenchen/fakultaeten-muenchen/fakultaet-gesundheit-und-pflege-muenchen/masterstudiengaenge-fakultaet-gesundheit-und-pflege-muenchen/angewandte-versorgungsforschung-msc/	first
Medizinische Hochschule Brandenburg Theodor Fontane	Versorgungsforschung	https://www.mhb-fontane.de/versorgungsforschung-studieren.html	first

*In the order they were found during the search.
