# Supplementary material for: A systematic mapping of public health master’s and structured doctoral programs in Germany
Source: BMC Med Educ. 2024 Aug 13;24:872. doi: 10.1186/s12909-024-05855-8 (PMC11323405; doi:10.1186/s12909-024-05855-8)
Supplement: Supplementary file 3 — Additional file 3. (Institution and title of doctoral programs, including website links) [file 12909_2024_5855_MOESM3_ESM.pdf]

Additional File 3 – Overview of mapped population health science and public health doctoral programs*

Institution:	Program title:	Program link:	Eligibility stage:
Heinrich-Heine-Universität Düsseldorf	Dr. PH (Public Health)	https://www.graduiertenzentrum-medizin.hhu.de/wegweiser/anmelden/dr-ph-public-health ; https://www.graduiertenzentrum-medizin.hhu.de/wegweiser/dazulernen/medizin/zahnmedizin-public-health-strukturiertes-ausbildungsprogramm (curriculum)	second
Medizinische Hochschule Hannover	Dr. Public Health	https://www.mhh.de/forschung/promotion-habil-apl/promotion-zum-dr-ph	second
Rheinische Friedrich-Wilhelms-Universität Bonn	Doctorate PhD (Public health, epidemiogy, health services research)	https://www.medfak.uni-bonn.de/de/fakultaet/karrierewege/nachwuchsfoerderung/phd ; https://iph.charite.de/en/academic_programs/phd_in_health_data_sciences/about_us/	second
Charité - Universitätsmedizin	Health Data Sciences	https://iph.charite.de/en/academic_programs/phd_in_health_data_sciences/ ; https://iph.charite.de/en/academic_programs/phd_in_health_data_sciences/about_us/ ; https://promotion.charite.de/en/procedure/regulations_2017/dissertation/	second
Ludwig-Maximilians-Universität München	Ph.D. Program - Medical Research in Epidemiology & Public Health	https://www.ibe.med.uni-muenchen.de/phd-studiengang/index.html ; https://www.en.mmrs.med.uni-muenchen.de/promotionsmoeglichkeiten_en/phd_en/index.html	second
University of Tübingen	PhD Program in Experimental Medicine	https://www.medizin.uni-tuebingen.de/en-de/medizinische-fakultaet/promotionen/phd-studiengang	second
Helmholtz Centre for Infection Research AND Hannover medical school AND Hannover biomedical research school	PhD Programme Epidemiology	https://www.helmholtz-hzi.de/en/career/phd-programme-epidemiology/objectives/	second
Universität Bielefeld	Public Health	https://www.uni-bielefeld.de/fakultaeten/gesundheitswissenschaften/studiengaenge/drph/index.xml	second
Helmholtz Graduate School Environmental Health (HELENA) & LudwigMaximilians-Universität München (LMU) & Technical University of Munich (TUM)	Helmholtz Graduate School Environmental Health (HELENA)	https://www.helmholtz-helena.de/training-program/training-and-education/index.html	first
Bonn international graduate school	Clinical and population science	https://bigs-clinpopscience.de/program-overview/	first
Universität Greifswald	Community Medicine	https://www.medizin.uni-greifswald.de/de/research-and-teaching/wissenschaftliche-graduierung/doctorate/phd-mdphd-und-dmdphd/	first
DKFZ International PhD Program in Heidelberg	Deutsches Krebsforschungszentrum -	https://www.dkfz.de/en/phd-program/	first

*In the order they were found during the search.

	"Cancer Risk Factors and Prevention" focus		
Medizinische Fakultät, Heinrich Heine, Universität Düsseldorf	Medicine PhD program with Health and Society focus	https://www.graduiertenzentrum-medizin.hhu.de/en/graduate-center-medicine/about-us/phd-programme; https://www.graduiertenzentrum-medizin.hhu.de/ueber-uns/phd-programm	first
Das Graduiertenprogramm der Medizinischen Fakultät der Universität des Saarlandes (UdS) AND Deutsche Hochschule für Prävention und Gesundheitsmanagement GmbH	Graduate program - (Dr. rer. med.)	https://www.dhfpg.de/graduiertenprogramm/promotionsvorbereitung-mit-system.html	first
Carl von Ossietzky University of Oldenburg	Graduate School Science, Medicine and Technology (OLTECH) - Medicine and Health Sciences	https://uol.de/en/oltech https://uol.de/en/oltech/phd-study-programmes/medicine-and-health-sciences	first
International Max Planck Research School	International Max Planck Research School for Population, Health and Data Science (IMPRS-PHDS)	https://www.imprs-phds.mpg.de/	first
Ludwig-Maximilians-Universität München, Germany	Ph.D. Program Medical Research - International Health	https://www.cih.lmu.de/education/study-programs/phd-program	first
Universitätsklinikum Hamburg-Eppendorf	PhD-Programm Nicht-Medizin	https://www.uke.de/forschung/promotion-phd-habilitation/phd-programm-nicht-medizin/index.html	first
Universität zu Lübeck - Das Zentrum für Bevölkerungsmedizin und Versorgungsforschung (ZBV)	Strukturierten Promotion in der Bevölkerungsmedizin und Versorgungsforschung	https://www.zbv.uni-luebeck.de/veranstaltungen/promotionskolleg	first

*In the order they were found during the search.
