# Supplementary material for: A systematic mapping of public health master’s and structured doctoral programs in Germany
Source: BMC Med Educ. 2024 Aug 13;24:872. doi: 10.1186/s12909-024-05855-8 (PMC11323405; doi:10.1186/s12909-024-05855-8)
Supplement: Supplementary file 4 — Additional file 4. (Extracted general data for population health science master’s programs) [file 12909_2024_5855_MOESM4_ESM.pdf]

Additional File 4 – General information on the stage-one eligible population health science (including public health) master's programs (general PHS program mapping)

| Institution and Program Title* | Title awarded: | Institution type: | Location: | Form of study: | ECTS: | Duration: (regular) | Duration: (max) |
|---|---|---|---|---|---|---|---|
| University Bielefeld<br><br>Public Health | M.Sc. | University | Bielefeld | full-time | 120 | 4 | NA |
| Charité AND Technical University of Berlin AND Alice Salomon Hochschule Berlin<br><br>Public Health | M.Sc.PH | Multiple (University AND Technical university AND university of applied science) | Berlin | both | 120 | 4 | 8 |
| Technical University of Dresden<br><br>Gesundheitswissenschaften - Public Health | M.PH | Technical university | Dresden | full-time | 120 | 4 | NA |
| APOLLON Hochschule der Gesundheitswirtschaft<br><br>Public Health - Prevention and Mental Health | M.Sc. | University of applied science | Online | both | 120 | 4 | 6 |
| Heinrich-Heine-Universität Düsseldorf<br><br>Public Health | M.Sc. PH | University | Düsseldorf | full-time (both) | 120 | 4 | 8 |
| Heinrich-Heine-Universität Düsseldorf AND Akademie für Öffentliches Gesundheitswesen<br><br>Public Health | M.Sc. PH/ÖG | University AND Academy | Düsseldorf | part-time | 60 | 3 | 8 |
| Jade Hochschule - Wilhelmshaven/Oldenburg/Elsfleth<br><br>Public Health | M.Sc. | University of applied science | Oldenburg | part-time | 90 | 5 | NA |
| FOM Hochschule für Oekonomie & Management - University of Applied Sciences<br><br>Public Health | M.Sc. | University of applied science | Multiple cities | part-time | 120 | 5 | NA |

*In the order they were found during the search.

| | | | | | | | |
|---|---|---|---|---|---|---|---|
| | | | | | | | |
| Hochschule Fulda - University of Applied Sciences<br><br>Public Health | M.Sc. | University of applied science | Fulda | both | 120 | 4 | 6 |
| Hochschule Fulda - University of Applied Sciences<br><br>Public Health Nutrition | M.Sc. | University of applied science | Fulda | both | 120 | 4 | 6 |
| Universität Bremen<br><br>Public Health - Gesundheitsförderung und Prävention | M.A. | University | Bremen | full-time | 120 | 4 | NA |
| Universität Bremen<br><br>Public Health - Gesundheitsversorgung, -ökonomie und -management | M.A. | University | Bremen | full-time | 120 | 4 | 4 |
| Technische Universität Chemnitz<br><br>Public Health mit Schwerpunkt Prävention und Evaluation | M.Sc. | Technical University | Chemnitz | full-time | 120 | 4 | 4 |
| IU Internationale Hochschule<br><br>Public Health (120) | M.Sc. | University of applied science | Online | both | 120 | 24 months | 48 months |
| IU Internationale Hochschule<br><br>Public Health (60) | M.Sc. | University of applied science | Online | both | 60 | 12 months | 24 months |
| Technische Hochschule Mittelhessen – THM<br><br>Public Health | M.Sc. | University of applied science | Gießen | full-time | 90 | 3 | NA |
| Hochschule für Angewandte Wissenschaften Hamburg<br><br>Public Health | MPH | University of applied science | Hamburg | both | 90 | 3 | NA |

*In the order they were found during the search.

| | | | | | | | |
|---|---|---|---|---|---|---|---|
| Ludwig-Maximilians-Universität München<br><br>Public Health | M.Sc. | University | Munich | full-time | 120 | 4 | NA |
| Medizinische Hochschule Hannover (MHH)<br><br>Bevölkerungsmedizin und Gesundheitswesen (Public Health) | M.Sc.PH | University of applied science | Hannover | both | 90 | 3 | NA |
| Universität Siegen<br><br>Digital Public Health | M.Sc. | University | Siegen | full-time | 120 | 4 | NA |
| Technische Hochschule Deggendorf<br><br>Global Public Health | M.Sc. | University of applied science | Pfarrkirchen | full-time | 90 | 3 | 3 |
| Leuphana Universität Lüneburg<br><br>Prävention und Gesundheitsförderung | MPH | University | Lüneburg | part-time | 60 | 4 | 4 |
| APOLLON Hochschule der Gesundheitswirtschaft<br><br>Public health - Umwelt & Gesundheit | M.Sc. | University of applied science | Online | both | 120 | 24 months (enrollment possible any time) | 32 months |
| Charité - Universitätsmedizin Berlin, Technische Universität Berlin, Alice Salomon Hochschule (Berlin School of Public Health)<br><br>Epidemiologie | M.Sc. | University of applied science, University, Technical university | Berlin | both | 60 | 2 | 4 |
| Universität Bremen<br><br>Epidemiologie | M.Sc. | University | Bremen | both | 120 | 4 | NA |
| Ludwig-Maximilians-Universität München<br><br>Epidemiologie | M.Sc. | University | Munich | full-time | 120 | 4 | 4 |
| Johannes Gutenberg-Universität Mainz<br><br>Epidemiologie (a - weiterbilend) | M.Sc. Epidemiology | University | Mainz | part-time | 60 | 4 | 8 |
| Johannes Gutenberg-Universität Mainz | M.Sc. Epidemiology | University | Mainz | full-time | 120 | 4 | NA |

*In the order they were found during the search.

| | | | | | | | |
|---|---|---|---|---|---|---|---|
| Epidemiologie (b - Konsekutiv) | | | | | | | |
| Charité - Universitätsmedizin Berlin<br><br>Applied Epidemiology | MSAE | University | Berlin | full-time | 120 | 4 | 4 |
| Hochschule Furtwangen - Informatik, Technik, Wirtschaft, Medien, Gesundheit<br><br>Angewandte Gesundheitsförderung | M.Sc. | University of applied science | Furtwangen | both | 90 | 3 | NA |
| Hochschule Ravensburg-Weingarten<br><br>Angewandte Gesundheitswissenschaft | M.A. | University of applied science | Weingarten | full-time | 90 | 3 | 3 |
| Westsächsische Hochschule Zwickau<br><br>Angewandte Gesundheitswissenschaft | M.Sc. | University of applied science | Zwickau | part-time | 120 | 6 | NA |
| Hochschule für Gesundheit - University of Applied Sciences<br><br>Angewandte Gesundheitswissenschaft | M.Sc. | University of applied science | Bochum | full-time | 120 | 4 | NA |
| Fachhochschule der Diakonie - Diaconia - University of Applied Sciences<br><br>Community Mental Health | M.A. | University of applied science | Bielefeld | part-time | 120 | 6 | NA |
| FH Münster University of Applied Sciences<br><br>Ernährung und Gesundheit | M.Sc. | University of applied science | Münster | full-time | 120 | 4 | NA |
| Hochschule für Gesundheit - University of Applied Sciences<br><br>Evidence-based Health Care | M.Sc. | University of applied science | Bochum | full-time | 90 | 3 | NA |
| Martin-Luther-Universität Halle-Wittenberg<br><br>Gesundheits- und Pflegewissenschaften | M.Sc. | University | Halle | full-time | 120 | 4 | NA |
| Universität zu Lübeck | M.Sc. | University | Lübeck | full-time | 120 | 4 | NA |

*In the order they were found during the search.

| | | | | | | | |
|---|---|---|---|---|---|---|---|
| Gesundheits- und Pflegewissenschaften | | | | | | | |
| Europäische Fachhochschule Rhein/Erft, european university of applied sciences<br><br>Gesundheitsforschung und Therapiewissenschaften | M.Sc. | University of applied science | Berlin, Online | part-time | 90 | 4 | NA |
| Hochschule für angewandte Wissenschaften Coburg<br><br>Gesundheitsförderung | M.Sc. | University of applied science | Coburg | full-time | 90 | 3 | NA |
| Pädagogische Hochschule Schwäbisch Gmünd<br><br>Gesundheitsförderung und Prävention | M.Sc. | University of applied science | Schwäbisch Gmünd | full-time | 120 | 4 | NA |
| Universität Erfurt<br><br>Gesundheitskommunikation | M.Sc. | University | Erfurt | both | 120 | 4 | NA |
| Pädagogische Hochschule Freiburg<br><br>Gesundheitspädagogik | M.A. | University of applied science | Freiburg | both | 120 | 4 | 6 |
| Hochschule Neubrandenburg - University of Applied Sciences<br><br>Gesundheitswissenschaften | M.Sc. | University of applied science | Neubrandenburg | full-time | 120 | 4 | NA |
| Westsächsische Hochschule Zwickau<br><br>Gesundheitswissenschaften | M.Sc. | University of applied science | Zwickau | full-time | 120 | 4 | NA |
| Hochschule Rhein-Waal - University of Applied Sciences<br><br>Gesundheitswissenschaften und -management | M.Sc. | University of applied science | Kleve | both | 90 | 3 | 6 |
| Universität Bayreuth<br><br>Gesundheitsökonomie | M.Sc. | University | Bayreuth | both | 120 | 4 | 8 |
| Universität zu Köln | M.Sc. | University | Köln | full-time | 120 | 4 | NA |

*In the order they were found during the search.

| | | | | | | | |
|---|---|---|---|---|---|---|---|
| Gesundheitsökonomie | | | | | | | |
| Hochschule Stralsund<br><br>Gesundheitsökonomie | M.Sc. | University of applied science | Stralsund | both | 90 to120 | 3 to 4 | 8 |
| APOLLON Hochschule der Gesundheitswirtschaft<br><br>Gesundheitsökonomie | M.A. | University of applied science | Online | both | 120 | 4(24 months) | 32 months |
| Universität Duisburg-Essen<br><br>Gesundheitsökonomie | M.Sc. | University | Essen | both | 120 | 4 | NA |
| Universität Bielefeld<br><br>Health Administration | M.A. | University | Bielefeld, online | part-time | 60 | 4 | NA |
| Hochschule Niederrhein<br><br>Health Care – Gesundheitswissenschaften | M.Sc. | University of applied science | Krefeld | both | 120 | 4 | 8 |
| Hochschule Osnabrück<br><br>HELPP - Versorgungsforschung und -gestaltung | M.Sc. | University of applied science | Osnabrück | full-time | 120 | 4 | NA |
| Universität Potsdam<br><br>Integrative Sport-, Bewegungs- und Gesundheitswissenschaft | M.Sc. | University | Potsdam | both | 120 | 4 | NA |
| Hochschule Braunschweig/Wolfenbüttel, Ostfalia Hochschule für angewandte Wissenschaften<br><br>Integriertes Versorgungsmanagement im Gesundheitswesen | M.A. | University of applied science | Wolfenbüttel | full-time | 120 | 4 | NA |
| Albert-Ludwigs-Universität Freiburg im Breisgau AND Hochschule Furtwangen<br><br>Interdisziplinäre Gesundheitsförderung | M.Sc. | University AND University of applied science | Freiburg, Furtwangen | part-time | 120 | 8 | NA |
| Charité Universitätsmedizin Berlin | MScIH | University | Berlin | both | 90 to 120 (for those with a | 2 | 8 |

*In the order they were found during the search.

| | | | | | | | |
|---|---|---|---|---|---|---|---|
| International Health | | | | | bachloer of only 180 ECTS vs 210) | | |
| Universität Trier<br><br>Interprofessionelle Gesundheitsversorgung | M.Sc. | Univerisity | Trier | full-time | 120 | 4 | NA |
| Carl von Ossietzky Universität Oldenburg<br><br>Versorgungsforschung | M.Sc. | University | Oldenburg | full-time | 120 | 4 | NA |
| Ruprecht-Karls-Universität Heidelberg<br><br>Versorgungsforschung und Implementierungswissenschaft im Gesundheitswesen | M.Sc. | University | Heidelberg | both | 120 | 4 | NA |
| Universität zu Köln<br><br>Versorgungswissenschaft | M.Sc. | University | Köln | full-time | 120 | 4 | NA |
| Universität Bremen<br><br>Community Health Care and Nursing: Versorgungsforschung und Versorgungsplanung | M.Sc. | University | Bremen | full-time | 120 | 4 | NA |
| Private Universität Witten/Herdecke gGmbH<br><br>Community Health Nursing | M.Sc. | University | Witten | full-time | 120 | 4 | NA |
| Europäische Fachhochschule Rhein/Erft, european university of applied sciences<br><br>Global Health | M.Sc. | University of applied science | Berlin, Online | part-time | 90 | 4 | NA |
| Rheinische Friedrich-Wilhelms-Universität Bonn<br><br>Global Health Risk Management & Hygiene Policies | M.Sc. | University | Bonn | full-time | 120 | 4 | NA |

*In the order they were found during the search.

| | | | | | | | |
|---|---|---|---|---|---|---|---|
| Albert-Ludwigs-Universität Freiburg im Breisgau<br><br>Global Urban Health | M.Sc. | University | Freiburg | full-time | 60 | 2 | NA |
| Universität zu Köln<br><br>Health Economics | M.Sc. | University | Köln | full-time | 120 | 4 | NA |
| Technische Universität München<br><br>Health Science - Prevention and Health Promotion | M.Sc. | Technical university | München | both | 120 | 4 | NA |
| Hochschule für Angewandte Wissenschaften Hamburg<br><br>Health Sciences | M.Sc. | University of applied science | Hamburg | both | 120 | 4 | NA |
| Ruprecht-Karls-Universität Heidelberg<br><br>International Health | M.Sc. | University | Heidelberg | both | 60 | 2 | 10 (5 years) |
| Ludwig-Maximilians-Universität München<br><br>International Health | MSc IH | University | München | full-time | 60 | 2 | 2 |
| Hochschule Fresenius<br><br>International Health Economics & Pharmacoeconomics | M.Sc. | University of applied science | Wiesbaden | full-time | 120 | 4 | NA |
| Katholische Stiftungshochschule für angewandte Wissenschaften München - Hochschule der Kirchlichen Stiftung des öffentlichen Rechts "Katholische Bildungsstätten für Sozialberufe in Bayern"<br><br>Angewandte Versorgungsforschung | M.Sc. | University of applied science | München | part-time | 90 | 4 | NA |
| Medizinische Hochschule Brandenburg Theodor Fontane<br><br>Versorgungsforschung | M.Sc. | University of applied science | Rüdersdorf | both | 120 | 4 to 5 (for part-time) | NA |

*In the order they were found during the search.
