# Supplementary material for: A systematic mapping of public health master’s and structured doctoral programs in Germany
Source: BMC Med Educ. 2024 Aug 13;24:872. doi: 10.1186/s12909-024-05855-8 (PMC11323405; doi:10.1186/s12909-024-05855-8)
Supplement: Supplementary file 5 — Additional file 5. (Extracted detailed data for public health master’s programs) [file 12909_2024_5855_MOESM5_ESM.pdf]

Additional File 5 – Detailed information on the stage-two eligible public health master's programs (in-depth program insights)

Institution and Program Title*	ECTS core:	ECTS elective:	ECTS for free electives:	Thesis Colloquium:	ECTS thesis:	Mandatory internship/ exchange:	Information about internship/ exchange:	Semester fees:	Associated university faculty:	Language of teaching:	Teaching mode:	Form of accreditation:	Accreditation provider:	Accreditation reviewer:	Membership to public health associations:
	ECTS from courses that all students must complete	*Total number of ECTS for required subjects that can be chosen often from a set of options*	*Number of ECTS for free choice subjects*	*Yes = when the colloquium consists of a seminar/or other form of semi regular support alongside the thesis (not colloquium as a examinations form)*	*ECTS for thesis (colloquium) - indicated ECTS for thesis colloquium should be recorded in brackets e.g., 24(6)*	*Yes/no*	*Yes/no, duration, semester number)*	*Student contribution fee, as/if listed on the website) Calculation of costs/ semester according to regular study time and using semester length of 6 months*	*E.g., medicine, public health, health science, ect.*	*Record primary language with secondary languages(s) in brackets, e.g. German (English*	*In presence, online or hybrid*	*Program or system - note should be looked up on separate website, please refer to technical guidelines*	*For current accreditation - note should be looked up on separate website, please refer to technical guidelines)*		
University Bielefeld<br><br>Public Health	42	48	0	yes	30	no	Not relevant	322,64€	Faculty of Health Sciences	German	In presence	System accreditation	Universität Bielefeld	Universität Bielefeld	ASPHER, dgph (via Bielefeld school of public health)
Charité AND Technical University of Berlin AND Alice Salomon Hochschule Berlin<br><br>Public Health	42	30	18 (6 could be internship or related work)	yes	24(6)	no	An "internship or professionally relevant student work can be claimed for up to 6 ECTS within 18 ECTS electives in the 3rd semester." This is possible but not madatory	approx. 300€	Berlin school of public health	German (English)	In presence	System accreditation	Charité	Charité	ASPHER, dgph (via Berlin school of public health)
Technical University of Dresden<br><br>Gesundheitswissenschaften - Public Health	75	15	0	yes	28(2)	no	Not relevant	300,30 €	Faculty of Medicine, the Department of Health Sciences / Public Health	German	In presence	System accreditation	Technische Universität Dresden	Technische Universität Dresden	ASPHER, dgph,
APOLLON Hochschule der	74	16	0	no	30	no	Not relevant	€ 259 /Monat =	Public Health and	German	online/distance learning	Program accredition	Akkreditierungsrat	AHPGS - Akkreditierungsagentur	

*In the order they were found during the search.

Gesundheitswirtschaft<br><br>Public Health - Prevention and Mental Health								1.554€/semester	Environmental Health		(with printed materials)			für Studiengänge im Bereich Gesundheit und Soziales	
Heinrich-Heine-Universität Düsseldorf<br><br>Public Health	90	0	0	yes	25(5)	yes, 30 Credits, 1-2 semesters, Alternatively, relevant professional experience of 900 hours can be recognized.	worth 30 ECTS, considered to be 900 contact hours. Relevant professional experience of 900 hours can also be recognised if it exceeds the 1 year work experience required for admission	8,000 euros for the entire further training course, which are charged during the course of study = 2000€/ semester	Faculty of Medicine (Center for Health and Society)	German (English)	In presence(block courses)	Program accreditation	Akkreditierungsrat	AQAS - Agentur für Qualitätssicherung durch Akkreditierung von Studiengängen	ASPHER, dgph
Heinrich-Heine-Universität Düsseldorf AND Akademie für Öffentliches Gesundheitswesen<br><br>Public Health	45	0	0	no	15	no	Not relevant	€2,903 (entire course) = 967,7€/ semester	Faculty of Medicine (Center for Health and Society)	German	In presence(block courses)	Program accreditation	Akkreditierungsrat	AQAS - Agentur für Qualitätssicherung durch Akkreditierung von Studiengängen	ASPHER, centre for health & society (chs), Akademie für Öffentliches Gesundheitswesen, dgph
Jade Hochschule - Wilhelmshaven /Oldenburg/Elsfleth<br><br>Public Health	62	10	0	no	18	no	Not relevant	378.22€	department "Technology and Health for People (TGM)	German	In presence (block courses) and online	Program accreditation	AHPGS - Akkreditierungsagentur für Studiengänge im Bereich Gesundheit und Soziales	AHPGS - Akkreditierungsagentur für Studiengänge im Bereich Gesundheit und Soziales	dgph
FOM Hochschule für Oekonomie & Management - University of Applied Sciences<br><br>Public Health	90	0	0	no	25	no	Not relevant	EUR 12,300.00 payable in 30 monthly installments of EUR 410 each Examination fee 500.00 euros Total costs 14,380.00	Health and Social Affairs	German	in presence	System accreditation	FOM Hochschule für Oekonomie & Management gGmbH	FOM Hochschule für Oekonomie & Management gGmbH	

*In the order they were found during the search.

								euros including enrollment fee, tuition fee and examination fee = 2.876€/ semester							
Hochschule Fulda - University of Applied Sciences<br><br>Public Health	60	30	0	no	30	no	Not relevant	319,39€	Departments Health science	German (English)	In presence	Program accreditation	Akkreditierungsrat	ACQUIN - Akkreditierungs-, Zertifizierungs- und Qualitätssicherungs-Institut	ASPHER, dgph
Hochschule Fulda - University of Applied Sciences<br><br>Public Health Nutrition	80	10	0	no	30	no	Not relevant	319,39€ (not found on website - however the fees across Fulda programs was implied to be the same)	Departments of Oecotrophology and Health Sciences - a joint degree program of the Departments of Nutritional, Food and Consumer Studies and Nursing and Health Care	German	In presence	Program accreditation	AHPGS - Akkreditierungsagentur für Studiengänge im Bereich Gesundheit und Soziales	AHPGS - Akkreditierungsagentur für Studiengänge im Bereich Gesundheit und Soziales	ASPHER, dgph
Universität Bremen<br><br>Public Health - Gesundheitsförderung und Prävention	81	0	9	yes	27(3)	no	An internship is a possibility for the 9 mandatory elective ECTS - decisions about internships however are made on a case by case basis	350€ (including semester ticket)	Faculty 11 - Human and Health Sciences	German	In presence	System accreditation	Universität Bremen	Universität Bremen	ASPHER, dgph
Universität Bremen<br><br>Public Health - Gesundheitsversorgung, -ökonomie und -management	81	0	9	yes	27(3)	no	An internship is a possibility for the 9 mandatory elective ECTS - decisions about internships	350€ (including semester ticket)	Faculty 11 - Human and Health Sciences	German	In presence	System accreditation	Universität Bremen	Universität Bremen	ASPHER, dgph

*In the order they were found during the search.

							however are made on a case by case basis								
Technische Universität Chemnitz<br><br>Public Health mit Schwerpunkt Prävention und Evaluation	75	15	0	no	30	no	Not relevant	295,18€ (included semester ticket)	Fakultät für Human- und Sozialwissenschaften	German	In presence	Are in the process of gaining system accreditation			dgph
IU Internationale Hochschule<br><br>Public Health (120)	70	20	0	yes	27(3)	no	Not relevant	From 316 to 529 euros per month, depending on the selected time model = min. 1.896€/semester max. 3.174€/semester	NA	German	online	System accreditation	IU Internationale Hochschule	IU Internationale Hochschule	
IU Internationale Hochschule<br><br>Public Health (60)	35	10	0	yes	13.5 (1.5)	no	Not relevant	From 448 euros per month, depending on the selected time model = appr. 2688€/semester	NA	German	online	System accreditation	IU Internationale Hochschule	IU Internationale Hochschule	
Technische Hochschule Mittelhessen – THM<br><br>Public Health	42	18	0	no	30	no	Not relevant	291,55€	Fachbereichs 05 Gesundheit	German	In presence	Program accreditation	Akkreditierungsrat	AHPGS - Akkreditierungsagentur für Studiengänge im Bereich Gesundheit und Soziales	
Hochschule für Angewandte Wissenschaften Hamburg<br><br>Public Health	60	0	0	no	30	no	Not relevant	€345 (semester contribution),2,400€ fees per semester, €7,200 (total fees)	Faculty of Life Sciences; Department of Health Sciences	English	In presence	System accreditation	Hochschule für Angewandte Wissenschaften Hamburg	Hochschule für Angewandte Wissenschaften Hamburg	ASPHER, dgph
Ludwig-Maximilians-Universität München<br><br>Public Health	90	30	0	yes	30	yes	Mandatory internship (24 ECTS) of at least 18 weeks is planned for	€152.30 (semester ticket included)	Medical faculty; The Institute for Medical Information	German	In presence	New accreditation in 2025	NA	AHPGS - Akkreditierungsagentur für Studiengänge im	ASPHER, dgph, EUGLOH – European University Alliance for

*In the order they were found during the search.

							the third semester of the Public Health master's degree → should give a concrete insight into the fields of activity of public health (cooperating research facilities and institutions). Possible in Germany or abroad.		Processing, Biometry and Epidemiology (IBE); The Pettenkofer School of Public Health (PSPH)					Bereich Gesundheit und Soziales	Global Health
Medizinische Hochschule Hannover (MHH)<br><br>Bevölkerungsmedizin und Gesundheitswesen (Public Health)	40	20	0	no	30	yes	Obligatory professional field internship over a period of 8 weeks (10 CP), accompanied by an introductory seminar on project management and phases of reflection. The internship can be carried out in all public health-related institutions in Germany and abroad.	390€ semester fees + 50€ per ECTS point (in total 90 ECTS = ~ 4,500 € in total and 1500€/semester	Institute for Epidemiology, Social Medicine and Health Systems Research at the Hannover Medical School	German	In presence	Program accreditation	AHPGS - Akkreditierungsagentur für Studiengänge im Bereich Gesundheit und Soziales	AHPGS - Akkreditierungsagentur für Studiengänge im Bereich Gesundheit und Soziales	dgph
Universität Siegen<br><br>Digital Public Health	72	18	0	no	30	no	Not relevant	326.50€	Lebenswissenschaftliche Fakultät	German (English)	In presence	System accreditation	Universität Siegen	Universität Siegen	
Technische Hochschule Deggendorf<br><br>Global Public Health	30	30	0	no	30	no	Not relevant	72€	Faculty European Campus Rottal-inn	English	In presence	Program accreditation	Akkreditierungsrat	ASIIN - Akkreditierungsagentur für Studiengänge der Ingenieurwissenschafte	

*In the order they were found during the search.

														n, der Informatik, der Naturwissenschaften und der Mathematik	
Leuphana Universität Lüneburg<br><br>Prävention und Gesundheitsförderung	35	10	0	no	15	no	Not relevant	10,800 euros, plus approx. 210 euros semester fee per semester, payment in installments possible = 2.910€/semester	Professional School (Berufsbegleitend)	German	In presence	Program accreditation	Akkreditierungsrat	FIBAA - Foundation for International Business Administration Accreditation	ASPHER, dgph
APOLLON Hochschule der Gesundheitswirtschaft<br><br>Public health - Umwelt & Gesundheit	74	16	0	no	30	no	Not relevant	€259/month = 6.216€ in total = 1.554€/semester	Public Health and Environmental Health	German	online/distance learning (with printed materials)	Program accreditation	Akkreditierungsrat	FIBAA - Foundation for International Business Administration Accreditation	

*In the order they were found during the search.
