# Supplementary material for: A systematic mapping of public health master’s and structured doctoral programs in Germany
Source: BMC Med Educ. 2024 Aug 13;24:872. doi: 10.1186/s12909-024-05855-8 (PMC11323405; doi:10.1186/s12909-024-05855-8)
Supplement: Supplementary file 6 — Additional file 6. (Extracted general data for population health science doctoral programs) [file 12909_2024_5855_MOESM6_ESM.pdf]

Additional File 6 – General information on the stage-one eligible population health science (incl. public health) doctoral programs (general PHS program mapping)

Institution and Program Title*	Title awarded:	Institution type:	Location:	Form of study:	ECTS:	Duration: (regular)	Duration: (max)
Heinrich-Heine-Universität Düsseldorf<br><br>Dr. PH (Public Health)	Dr. PH	University	Düsseldorf	NA	NA	NA	4 years
Medizinische Hochschule Hannover<br><br>Dr. Public Health	Dr. PH	University	Hannover	NA	no ECTS	NA	NA
Rheinische Friedrich-Wilhelms-Universität Bonn<br><br>Doctorate PhD (Public health, epidemiogy, health services research)	Ph.D.	University		NA	NA	3 years	NA
Charité - Universitätsmedizin<br><br>Ph.D. Program - Medical Research in Epidemiology & Public Health	Ph.D.	University	Berlin	full-time	180 (30 from coursework)	3 years (6 semesters)	9 semester (with adequate reasons, e.g. pregnancy, childcare, longer term health condition, etc.)
Ludwig-Maximilians-Universität München<br><br>PhD Program in Experimental Medicine	Ph.D.-EPH	University	Munich	full-time	180 (30 from coursework)	3 years (to 5 years; 6 to 10 semesters according to program flyer)	NA
University of Tübingen<br><br>PhD Program in Experimental Medicine	Ph.D.	University	Tübingen	full-time (implied)	30 (coursework only)	3 years	4 years
Helmholtz Centre for Infection Research AND Hannover medical school AND Hannover biomedical research school<br><br>PhD Programme Epidemiology	Ph.D. or Dr. rer. nat.	Research institution	Braunschweig	NA	180 credit points - not ECTS (55 of coursework) - points are determined by the number of dedicated hours	3 years	4 years
Universität Bielefeld<br><br>Public Health	DrPH or PhD in Public Health	University	Bielefeld	full-time	NA	3 years	3 years

*In the order they were found during the search.

Helmholtz Graduate School Environmental Health (HELENA) & Ludwig Maximilians-Universität München (LMU) & Technical University of Munich (TUM)<br><br>Helmholtz Graduate School Environmental Health (HELENA)	Dr. rer. nat. OR PhD OR Dr. rer. biol. hum.	Research institution AND Universities	Munich	full-time	uses own working hours system to determine work load distribution	3 years	4 years
Bonn international graduate school<br><br>Clinical and population science	Ph.D. OR MD/PhD OR Dr. troph.	Graduate school through University	Bonn	NA	NA	3 years	NA
Universität Greifswald<br><br>Community Medicine	Ph.D. OR MD/PhD OR DMD/PhD	University	Greifswald	Not fixed can be part or full time - usually based on a contract of <26 hrs per week / 65%	24 (Course work)	3 years	No maximum
DKFZ International PhD Program in Heidelberg<br><br>Deutsches Krebsforschungszentrum - "Cancer Risk Factors and Prevention" focus	Ph.D. OR Dr. rer. nat. OR Dr. sc. Hum OR Dr. med.	Research institution AND University	Heidelberg	NA	NA	3 to 4 years	NA
Medizinische Fakultät, Heinrich Heine, Universität Düsseldorf<br><br>Medicine PhD program with Health and Society focus	Ph.D. in Medical Sciences OR Dr. rer. med.	University	Düsseldorf	No information provided also after contact	no ECTS - usually 300 hours of course work	3 to 4 years	5 years
Das Graduiertenprogramm der Medizinischen Fakultät der Universität des Saarlandes (UdS) AND Deutsche Hochschule für Prävention und Gesundheitsmanagement GmbH<br><br>Graduate program - (Dr. rer. med.)	Dr . rer. med.	University AND University of applied science	Saarbrücken	both	120	2 years	NA
Carl von Ossietzky University of Oldenburg	Dr. rer. medic.	University	Oldenburg	both (full-time and part-time)	30	3 years (6 semesters)	no maximum

*In the order they were found during the search.

Graduate School Science, Medicine and Technology (OLTECH) - Medicine and Health Sciences							
International Max Planck Research School<br><br>International Max Planck Research School for Population, Health and Data Science (IMPRS-PHDS)	Ph.D.	Research institution (AND University)	Rostock	full-time	ECTS not used	3 years	extension possible with funding and good reason
Ludwig-Maximilians-Universität München, Germany<br><br>Ph.D. Program Medical Research - International Health	Ph.D. in Medical Research – International Health	University	Munich	full-time	180	3 years	5 years
Universitätsklinikum Hamburg-Eppendorf<br><br>PhD-Programm Nicht-Medizin	Ph.D.	University	Hamburg	NA	180 (30 from coursework)	3 years	NA

*In the order they were found during the search.
