# Supplementary material for: A systematic mapping of public health master’s and structured doctoral programs in Germany
Source: BMC Med Educ. 2024 Aug 13;24:872. doi: 10.1186/s12909-024-05855-8 (PMC11323405; doi:10.1186/s12909-024-05855-8)
Supplement: Supplementary file 7 — Additional file 7. (Extracted detailed data for public health doctoral programs) [file 12909_2024_5855_MOESM7_ESM.pdf]

Additional File 7 – Detailed information on the stage-two eligible public health doctoral programs (in-depth program insights)

**Institution and Program Title***	**ECTS core:**	**ECTS elective:**	**Mandatory internship/ exchange:**	**Information about internship/exchange:**	**Semester fees:**	**Associated university faculty:**	**Language of teaching:**	**Teaching mode:**	**Form of accreditation:**
	ECTS from courses that all students must complete	*Mandatory electives are subjects that are mandatory, but self selected, often from a variety of options*			*As listed on program website*			*In presence, online or hybrid)*	
Heinrich-Heine-Universität Düsseldorf<br><br>Dr. PH. (Public Health)	NA<br>Courses offered, no reference to credit system	NA<br>No credits for elective courses indicated	No	NA	NA	Faculty of Medicine	German	In presence	NA
Medizinische Hochschule Hannover<br><br>Dr. Public Health	NA<br>Courses require, no reference to credit system	None	No	Not mandatory**	Usual semester fees**<br><br>Approx. 390€	The medical university	German & English	In presence	None**
Rheinische Friedrich-Wilhelms-Universität Bonn<br><br>Doctorate Ph.D. (Public health, epidemiogy, health services research)	NA<br>Courses require, ECTS not fixed**	At least 2 research -related courses (i.e. 2 course units per week)**	No	NA	NA	Faculty of Medicine	German & English	In presence	NA
Charité - Universitätsmedizin<br><br>Ph.D. Program - Medical Research in Epidemiology & Public Health	18	12	No	If suitable, possible	free of charge with the exception of the Charité enrollment fee, option to apply for scholarships by DAAD	The medical university	English	In presence	NA
Ludwig-Maximilians-Universität München<br><br>Ph.D. Program in Experimental Medicine	0	30	No	None**	LMU enrolment fees approx. 150€	Faculty of Medicine	English	In presence or online (some courses)	None**
University of Tübingen<br><br>PhD Program in Experimental Medicine	8	22	No	NA	None. A prerequisite for the application is the commitment of a university professor of the Medical Faculty Tübingen to supervise the	Faculty of Medicine	English	In presence	NA

*In the order they were found during the search.
**Answers retrieved via email request.

					doctorate and funding.				
Helmholtz Centre for Infection Research AND Hannover medical school AND Hannover biomedical research school<br><br>Ph.D. Program Epidemiology	2 parts: individual epidemiological research activity (125 Credit Points) + mandatory educational program (55 Credit Points, minimum 300 Teaching Units (TU's 45 min each))	NA	No	Appreciated and are supported	€ 355,42 + Current admissions for the PhD Program are only possible if funding is assured by the applicant (e.g. by a scholarship or a third party).	Department of Epidemiology and International Graduate School for Infection Research, Hanover Biomedical Research School (HBRS)	English	In presence	NA
Universität Bielefeld<br><br>Public Health	NA	NA	No	NA	NA	Faculty of Health Science	German	In presence	NA

**Institution and Program Title***	**Accreditation provider:**	**Membership to public health associations:**	**Core curriculum:**	**Dissertation type:**	**Requirements for publications:**	**Mentoring offered:**
			Yes/No	*Monography, cumulative or self-selection*	*Number and type of paper, accepted ranking of journal etc.*	*Yes/no - Mentors being an individual that is not directly related to supervision, but that offers academic support of Ph.D. students*
Heinrich-Heine-Universität Düsseldorf<br><br>Dr. PH. (Public Health)	NA	NA	Yes	Self-selection	At least undivided first authorship of an original work published or accepted for publication in an internationally recognized journal listed on PubMed or ISI Web of Knowledge that has a peer review process.	NA
Medizinische Hochschule Hannover<br><br>Dr. Public Health	None**	German Society for Public Health (DGPH), the German Society for Social Medicine and Prevention (DGSMP) and the European Public Health Association (EUPHA)**	Yes	Self-selection Option 1: monograph and Option 2: Publication-based dissertation - a cumulative dissertation<br>a scientific paper (dissertation) written in German or English	Internationally recognized scientific journals with a peer review system, generally no more than three years old. The doctoral candidate must be the sole or first author or equal first author in one of these publications.	Program from Bosch Stiftung --> "includes intensive mentoring", other candidates at medical faculty "attend a series of courses, make formal presentations, and participate in a regular doctoral colloquium"**

*In the order they were found during the search.
**Answers retrieved via email request.

Rheinische Friedrich-Wilhelms-Universität Bonn<br><br>Doctorate Ph.D. (Public health, epidemiogy, health services research)	NA	NA	No	Self-selection- Option 1: monography. Option 2: Publication-based dissertation	Three major original academic publications (publication doctoral thesis) with related contents that have been accepted for publication by international journals and show the doctoral student named as first author at least once are equivalent to this doctoral thesis. At least one of the publications should be no more than one year old at the time the doctoral examination procedure is opened. The publications must clearly indicate that they originate from the University of Bonn.	“Each student has its supervisor (and 3 other members within a thesis committee)”**
Charité - Universitätsmedizin<br><br>Ph.D. Program - Medical Research in Epidemiology & Public Health	NA	ASPHER (for the Berlin school of Public health. No direct mention of PhD programs)	No (there are predefined ratio of ECTS required from certain subjects, but not a predefined range of courses actually required by all students)	Cumulative	Should be prepared in English. The publication date of the most recent publication may not be more than one year before initiation of the doctoral examination procedure.<br><br>Must include at least one original publication by the candidate as lead author in an international "peer-reviewed" journal or three original publications, with at least one by the candidate as lead author, in a "peer-reviewed" journal<br><br>In case of an original publication as first author in a leading international peer-reviewed journal, up to two additional original publications in peer-reviewed journals can be submitted. These journals do not have to be internationally leading journals. First author or co-authorship possible.	NA
Ludwig-Maximilians-Universität München<br><br>Ph.D. Program in Experimental Medicine	None**	ASPHER (Pettenkofer School of Public Health Munich, PhD program not directly mentioned)	Yes	Self-selection	Cumulative: It must consist of at least 2 articles, which have been published in or accepted for publication by a peer-reviewed journal. The Ph.D. candidate must be first author of at least one article	"Our Ph.D. students are being supervised during their Ph.D. journey by a TAC Thesis Advisory Committee, details you can find here."**
University of Tübingen<br><br>PhD Program in Experimental Medicine	NA	NA	Yes	Self-selection	The dissertation must be written in English. Scientific progress must be recognizable, and the most important results of the work should be published or accepted for publication in the form of one or more original articles in high-ranking, English-language scientific journals. The PhD Board may set up additional criteria for the form and scope of a dissertation.	NA

*In the order they were found during the search.
**Answers retrieved via email request.

Helmholtz Centre for Infection Research AND Hannover medical school AND Hannover biomedical research school<br><br>Ph.D. Programme Epidemiology	NA	NA	Yes	Self-selection	Alternatively (instead of a Monograph), usually two first author publications in internationally peer reviewed science journals (published or accepted) as a cumulative thesis. Shared first authorships are allowed. The PhD student's personal contribution to such publications must be clearly identified as well as the contribution of the other authors. In that context, "accepted" shall be deemed equivalent to "published". As for this publication requirement, exceptions are possible with reasons to be given by the supervisor.	NA
Universität Bielefeld<br><br>Public Health	NA	ASPHER (and names Dr.PH)	Yes	Self-selection (usually a monography)	Instead of an individual work, the following can also be submitted:<br>1. A cumulative dissertation comprising at least four manuscripts, of which at least 2 are first authors. One of the first authorships and a total of two of the four manuscripts must be in English. At the time the doctoral procedure begins, all manuscripts must be submitted demonstrably in a peer-reviewed journal listed in relevant scientific databases publication to be accepted. The candidate's share of the manuscripts in joint authorship must be evident from the paper. The authorship of the individual parts is from to be confirmed in writing by the candidate and the co-authors. Overall, this form of the dissertation must have at least the academic rank of an individual work. For the cumulative dissertation, the academic context of the individual manuscripts is the responsibility of the candidate<br>Present candidates in a synopsis of 20 to 40 pages of 2000 characters each.	NA

**Institution and Program Title**	**Organisation of Supervision:**<br><br>(individual supervisor, supervisor committee, etc.)
Heinrich-Heine-Universität Düsseldorf	§ 5 "Supervision of the doctorate<br>(1) The doctorate of a doctoral candidate is accompanied by a specialist supervisor (hereinafter referred to as supervisor) and at least one other competent scientist (hereinafter referred to as co-supervisor). The supervisors are appointed by the dean. The work of the doctoral candidate should be carried out in constant consultation with the supervisors.

*In the order they were found during the search.
**Answers retrieved via email request.

Dr. PH. (Public Health)	(2) The supervisor must belong to the medical faculty or another faculty of the Heinrich-Heine-University Düsseldorf or a partner institution. All supervisors (supervisors and co-supervisors) must belong to the group of university teachers (including extracurricular professors, junior professors/junior professors, private lecturers or equivalent qualifications). At least one supervisor must work full-time at the medical faculty of Heinrich Heine University.<br>(3) The co-supervisor must be independent of the supervisor and may not be subordinate to him/her under employment law or belong to the same department. His/her task is the additional supervision of the doctoral candidate.<br>(4) In justified cases, the co-supervisor can be changed, taking into account § 5 (1) sentence 1 and sentence 2. The change must be reported to the dean immediately by the supervisor<br>and the doctoral candidate can be applied for. This request must include the following in particular<br>Information includes:<br>a) the name of the previous co-supervisor;<br>b) the name of the future co-supervisor and a statement from this person confirming their willingness to take on co-supervision.<br>c) a comprehensible reason for the change.<br>(5) Before starting work on the dissertation, the doctoral candidate conducts a consultation with the supervisor and the co-supervisor. Others can also participate in this conversation<br>people participate. As a result of the consultation, the doctoral candidate concludes a written supervision agreement (according to § 67 (2) HG NRW) with the supervisor, in which claims,<br>Rights and obligations of both sides are clearly defined.<br>The supervision agreement is signed by everyone who took part in the consultation. Each of these persons and the medRSD office receive a copy of the signed supervision agreement.<br>(6) The doctoral candidate writes a progress report at least once a year. While working on the dissertation, the doctoral candidate, the supervisor and the co-supervisor meet at least once a year to discuss this progress report together. Other people can also attend this meeting. The progress report is signed by everyone who attended the counseling session. Each of these persons and the medRSD office will receive a copy of the progress report." (translated from orginal found in Promotionsordnung)
Medizinische Hochschule Hannover<br><br>Dr. Public Health	At the stage of aplication to PhD the selected supervisor and a second supervisor must be named. The topic, the objective, the planned investigations and the expected new scientific findings must be presented and the chosen supervisor and a second supervisor must be named. In the case of external dissertations, the cooperative character (§ 5 Para. 3) of the work must also be stated. All supervisors must confirm the registration by signature, declaring that they will supervise the scientific project and review the dissertation. The first supervisor must inform the respective head of department by sending a copy of the dissertation notification. The preparation of the dissertation must be scientifically supervised (supervisor) by a member of the teaching staff employed at the MHH (internal teaching staff). The supervisor must have publications in the field of the dissertation topic to be awarded. In addition, a second supervisor must be named who is a member of the teaching staff in another department of the MHH. In justified exceptional cases, the second supervisor may be a member of the teaching staff of another university. In addition, the doctoral committee may approve exceptions, such as supervision by persons with qualifications comparable to the habilitation. Postdoctoral researchers who have not yet habilitated can be appointed as junior supervisors. A structured discussion takes place regularly at least once a year between all the supervisors involved and the doctoral candidate in order to agree on the annual schedule. The jointly agreed target agreements are documented by the participants. (2) Doctoral candidates who transfer with their supervisor to Hannover Medical School may be approved by the Doctoral Commission with corresponding adjustment of § 1, Para. 3, and § 5, Para. 1, to the doctoral period still required. The regulations of § 6 remain unaffected. (3) In addition to providing professional advice, the supervisors also have the task of evaluating the project presentations (§ 6, Para. 2). (4) If a dissertation is written outside the MHH (external dissertation), scientific cooperation with the corresponding external institution must be convincingly presented in the dissertation announcement for the doctoral committee. In addition to the internal first and second supervisor, an external supervisor must be chosen. The external supervisor must have a habilitation or a qualification comparable to a habilitation. (5) If the supervisory relationship is terminated before the doctoral application is submitted, the doctoral candidate and all supervisors must inform the President of the Hannover Medical School immediately in a joint letter, stating the reasons.
Rheinische Friedrich-Wilhelms-Universität Bonn<br><br>Doctorate Ph.D. (Public health, epidemiogy, health services research)	§3 "The first reviewer is the professor responsible under the supervision agreement for supervision of the doctoral student. The second reviewer must represent the discipline of the doctorate. The second reviewer must not belong to the working group, institute or clinic of the responsible professor. (4) At least two of the members, including one reviewer, must be full-time professors in the Faculty of Medicine at the University of Bonn. One reviewer should be a professor outside the Faculty of Medicine of the University of Bonn with an international reputation in the area of the doctoral thesis."
Charité - Universitätsmedizin<br><br>Ph.D. Program - Medical Research in Epidemiology & Public Health	§7 Scientific research work (1) The scientific research work with the aim of preparing a dissertation serves as proof of the ability to carry out independent scientific work. (2) The technical supervision of the dissertation is carried out by an individually composed supervisor committee ("thesis committee") consisting of three supervisors. As a rule, at least one of the supervisors should come from the field of biostatistics, epidemiology, meta research or population health science. For the rest, reference is made to the provisions of the applicable doctoral degree regulations for the supervision of doctoral projects. The supervisor committee is formed at the beginning of the project in agreement between the student and the PhD committee. (3) The composition of the supervisory board can be changed for technical or non-technical reasons by mutual consent of those involved and the PhD committee. (4) As part of the scientific research work, research stays at suitable research institutions in Germany and abroad are possible. Location, frequency and duration depend on the concrete progress made in each case and the suitability for the topic of the dissertation. (5) The supervisory committee and the doctoral project are documented in a doctoral agreement and registered with the doctoral office of the Charité - Universitätsmedizin Berlin during the mandatory registration of doctoral projects.
Ludwig-Maximilians-Universität München	"Supervisor

*In the order they were found during the search.
**Answers retrieved via email request.

Ph.D. Program in Experimental Medicine	The doctoral candidate is supervised by an authorized person (supervisor), who can be a member of the Medical Faculty, or from another faculty at the LMU or can belong to an external institution. Please note that for students in the individual PhD the supervisor must be a member of the LMU Medical Faculty.<br><br>Supervision agreement<br><br>Please have your supervisor sign your supervision agreement, which is generated as a PDF by the Campus Portal, with an original, handwritten signature. Sign it yourself by hand as well. Upload a scan of the signed supervision agreement to your Campus Portal account and send the original by mail to the MMRS (Bavariaring 19, 80366 München).<br><br>Thesis Advisory Committee (TAC)<br><br>During the first semester of the doctoral research project, each Ph.D. candidate must have a Thesis Advisory Committee (TAC). The TAC is comprised of the supervisor, the second reviewer (must be private lecturer or professor) and a third TAC member (has to be at least an independent group leader). All areas of the research project should be appropriately covered. At least two members of the TAC must be members of the LMU's Medical Faculty.<br><br>The TAC has to fulfill the following criteria:<br><br>The second TAC member belongs to another department than your main supervisor. TAC member 1 and 2 must be a private lecturer or professor. TAC member 3 should to be at least an independent group leader (PI). Two out of three TAC members must be part of the Medical Faculty for candidates in the individual PhD: TAC member 1 has to be part of the Medical Faculty, 2 OR 3 can be from outside of the Medical Faculty (e.g. another department, another LMU faculty or another university)" (https://www.en.mmrs.med.uni-muenchen.de/promotionsmoeglichkeiten_en/phd_en/betreuung-phd_en/index.html)
University of Tübingen<br><br><br>PhD Program in Experimental Medicine	"Section 5 Supervision<br><br>(1) The primary supervisor must be a member of the faculty with the rank of Junior professor, Professor, Hochschuldozent or Privatdozent. As a rule, he is the leader of the work group in which the student's research project will be carried out. The PhD student and the primary supervisor conclude a Betreuungsvereinbarung (supervision agreement) following the template provided by the Faculty of Medicine.<br><br>(2) The PhD Board appoints a doctoral committee for each PhD student consisting of the primary supervisor and two additional members with expertise in the area of the dissertation. At least one member of the doctoral committee must have the rank of full-time professor at the Faculty of Medicine. Professors, including those from Fachhochschulen, junior professors, retired and emeritus professors, Privatdozenten, honorary professors and guest professors, qualified members of non-university research institutes and foreign universities and, after decision on an individual basis, experienced scientific staff with doctoral degrees are eligible for appointment as members of the doctoral committee.<br><br>Regulations for the PhD-Degree Program Experimental Medicine<br><br>(3) If the candidate received his degree in a subject that is part of another faculty at the University of Tübingen, one of the three members of the doctoral committee must be appointed from that faculty or department. That member shall be recommended by the dean of the respective faculty.<br><br>(4) The PhD Board may delegate the task of appointing the doctoral committee to the Chairperson of the PhD Board.<br><br>(5) The doctoral candidate shall make regular reports (at least once a year) on the progress of his doctoral project to the doctoral committee in a joint meeting. The doctoral committee advises the PhD student with regard to the volume and content of the doctoral studies and, where appropriate, additional requirements, discusses the PhD student's research proposal with him, submits written comments on the PhD student's preliminary reports (Section 6 paragraph 3) and, together with additional examiners, forms the examination board for the oral examination (Section 10 paragraph 2)."<br><br>The doctoral supervisor may be heard when there are questions pertaining to his advisee's case. Additional experts may be consulted if special questions arise. (section 3)<br><br>Together with the PhD student, the supervisor shall work out a written outline of the goals and execution of the planned project before work is actually taken up. The outline includes the written confirmation that the PhD student was made aware of the principles of good scientific practice by his supervisor. If in the course of doctoral work a conflict between student and supervisor should arise, the Dean or the Chairperson of the PhD Board may be consulted as mediators. Doctoral proceedings may be conducted in joint supervision with a foreign university if a corresponding agreement has been signed with the foreign university and approved by the PhD Board. Doctoral proceedings may be conducted in joint supervision with a foreign university if a corresponding agreement has been signed with the foreign university and approved by the PhD Board. The candidate is supervised by one academic faculty member of each of the two participating universities. The supervisor from the foreign university is appointed as second reporter in the Tübingen doctoral proceedings. If he is prevented from acting as second reporter, another member of the foreign university shall be nominated by that university PhD students shall develop an individual study program in the framework of the above-mentioned curriculum.

*In the order they were found during the search.
**Answers retrieved via email request.

Helmholtz Centre for Infection Research AND Hannover medical school AND Hannover biomedical research school<br><br>Ph.D. Program Epidemiology	"§ 6<br><br>Supervision<br><br>(1) PhD students shall supervised by the members of their respective thesis advisory board (§ 4) appointed by the PhD Program Committee. The responsibilities of the team shall be:<br><br>a) To act as co-supervisors and to give individual expert advice to PhD students all through their PhD studies.<br><br>b) Within the scope of their research project, students have to work with appropriate methods on a clearly defined subject so that, with some realistic prospect of success, scientific knowledge can be expected to be incremented and the results of such research should be published in international peer-review journals. The co-supervisors shall make sure, and satisfy the PhD Program Committee to that effect, that students are not entrusted with any tasks unrelated to their PhD studies.<br><br>c) To evaluate PhD students' progress during their studies by receiving their reports (annually) and conducting exams; and to assess their written final examination papers. The thesis advisory board meeting is conducted at least once a year. It is documented by a written protocol.<br><br>d) Within a time of probation of 6 months from start of the PhD project, PhD students have to prove themselves and are evaluated mainly by the main supervisors. Within this time peroid, student status can be changed easily on both sides in agreement with the team of co-supervisors and PhD Program Committee. Upon request, the PhD Program Committee can decide about the termination of collaboration with the student. The termination of collaboration after the time of probation requires first a moderated discussion by a member of the PhD Program Committee between the student and the respective thesis advisory board. A student member of the PhD Program Committee is allowed to join as well. Afterwards, the PhD Program Committee announces their recommendations.<br><br>(2) The supervisors shall be responsible for the financing of the respective research project and shall make efforts, during the standard period of PhD studies (three years), to raise the money needed for the PhD students they are in charge of. Any scholarships available at the MHH shall be awarded or distributed to the individual PhD programs by resolution of the HBRS Committee of MHH.<br><br>(3) (Co-)supervisors should assist PhD students in planning their further professional career.<br><br>(4) The responsibilities of (co-)supervisors for PhD students shall end upon the date when the latter pass their PhD examination (§ 10), which is normally three years but no later than five years after commencement of PhD studies. The duration of PhD could only be extended in exceptional cases for a maximum of one year. Reasons could be: a) intermittent medical training (specialization) by medical students during their PhD studies, b) prolonged parental leave or c) serious illness." (from MHH - promotionordnung - as they are the institution responsible for issuing the PhD)
Universität Bielefeld<br><br><br>Public Health	“6. Supervision (§ 7 RPO)<br><br>(1) The doctoral committee appoints a first supervisor in agreement with the candidate or a first supervisor and a second supervisor for the dissertation. The second supervisor or the second supervisor is usually appointed within one year of the acceptance of the doctoral candidate. The first supervisor and the second supervisor must be eligible to vote, be members of the group of university teachers of the faculty or other qualified members of the faculty who are eligible to vote. In special cases, members of the faculty who have a doctorate and who have been doctorate have been employed in higher education for at least six years, be second supervisors, provided that the respective working group leader agrees to this project. Besides can also members of the faculty with a doctorate who have themselves acquired doctoral positions financed from third-party funds and intend to fill these positions with individuals who in turn are pursuing doctoral studies at the faculty who Apply for initial supervision of these people in writing, provided that the respective work group leader agrees to this plan. The doctoral committee verifies that the requirements are met and decides on the recognition of a person according to sentence 4 or 5 as the first supervisor. will be one If the first supervisor is appointed according to sentence 4 or 5, the second supervisor must be present be a voting member of the group of professors of the faculty. leaves one supervisor, or if he or she retires, he or she retains the right to to complete the supervision of the doctorate that has been started and to be appointed as an assessor for it. She or he is considered a member of the faculty for this procedure. Exceptional cases include emeritus or retirement transferred university teachers also entitles new doctoral students to be supervised if the topic of the dissertation is no longer represented by members of the faculty. (2) For interdisciplinary work, it is possible to employ university teachers or private lecturers Private lecturers and other habilitated members of other faculties or other universities as second supervisors or to appoint second supervisors if they have a particularly good academic record in the subject area of the dissertation. In this case, a justified written application for supervision of a candidate is required candidates. The doctoral committee decides on the application. For cooperative forms of care, orientation is based on the recommendations of the DFG."

*In the order they were found during the search.
**Answers retrieved via email request.
