# Supplementary material for: A systematic mapping of public health master’s and structured doctoral programs in Germany
Source: BMC Med Educ. 2024 Aug 13;24:872. doi: 10.1186/s12909-024-05855-8 (PMC11323405; doi:10.1186/s12909-024-05855-8)
Supplement: Supplementary file 8 — Additional file 8. (Extracted data on competencies aimed at by public health master’s and doctoral programs) [file 12909_2024_5855_MOESM8_ESM.pdf]

Additional File 8 – Data extraction competencies for public health master's and doctoral programs

Competencies master's programs

University Bielefeld: Public Health

"...Public Health course is science-oriented, multidisciplinary and professionally oriented. In the courses, you will acquire specialist knowledge and analytical and methodical skills in the broad spectrum of public health, such as on health policy reform processes, health and care system development and health psychology and sociology.
The course of study is divided into a technical basis in which you gain a basic understanding of health science fields of action and working methods, followed by an elective area in which you deepen your knowledge in one of two profiles.
As part of the profile "In-depth interdisciplinary content and approaches to public health" you will gain insights into the classic disciplines of public health. The "International Perspectives of Public Health" profile gives you the opportunity to set an international focus during your course of study and thereby do justice to the growing importance of international aspects and topics in public health." (From Website)
"The master's program is intended to provide students with specialist knowledge, scientific skills and methodological skills as well as job-related qualifications, taking into account the interdisciplinary, international and research-oriented profile of Bielefeld University. The aim of the course is to deepen and expand specialist knowledge, skills and competencies that have already been acquired, to independently acquire and apply knowledge and to be able to make scientifically sound decisions, taking into account social, scientific and ethical findings that result from the application of the knowledge and result from the decisions...(3) The guiding principle for teaching at Bielefeld University is the scientific-academic character of the course, which imparts a wide range of qualifications. The students should be enabled to set their own priorities, to develop independent questions and positions, and also to get to know technical and interdisciplinary perspectives that correspond to the complexity of current and future professional challenges. Bielefeld University expects and encourages active study." (From Examination regulations)

Charité AND Technical University of Berlin AND Alice Salomon Hochschule Berlin: Public Health

"…The Master's degree in Public Health enables:

- to analyze and evaluate the health situation of the population as a whole and of specific populations at municipal, regional and national level as well as in international comparison;
- to determine the physical, psychological, social and environmental conditions of health and disease and their interaction;
- to analyze and evaluate the structure, cost development and dynamics in healthcare systems;
- to evaluate care structures and services as well as prevention and health promotion programs;
- for the planning, implementation and evaluation of measures for health promotion and prevention as well as for curative, rehabilitative, nursing and palliative care.
- to identify public health problems and tasks and to independently develop and formulate scientifically based solutions;

- on the systematic consideration of gender and social differences in public health". (From ASH page)

Technical University of Dresden: Gesundheitswissenschaften - Public Health

"…The aim of the Public Health Master's programme at the Faculty of Medicine of TU Dresden is to further train and qualify students for tasks in research, planning and management in national and international health systems.
After completing the degree programme, students will have a broad knowledge of the structures and tasks of health and social services as well as of the scientific foundations for disease prevention and health promotion. They will be able to scientifically analyse public health problems and to develop well-founded proposals for solutions. In addition, they will be qualified for advanced scientific work (e.g. doctorate Dr. rer. medic.)." (From website and flyer)

APOLLON Hochschule der Gesundheitswirtschaft: Public Health - Prevention and Mental Health

"…The course provides you with all the skills you need to analyze conditions for mental health and illness and, on this basis, to develop effective prevention approaches involving various disciplines. You can also evaluate the projects afterwards. Specifically, you acquire:

- Competences in health psychology including neuropsychological aspects of health behaviour.
- Sound specialist knowledge of prevention and mental health, e.g. B. in children, young people, employed and older people.
- Knowledge in the field of international health policy and global public health.
- Specific methodological know-how for health research as well as medical and health scientific writing.
- Competencies in public health ethics and care management."

(From Website)

Heinrich-Heine-Universität Düsseldorf: Public Health

"…Since 1991, the Düsseldorf continuing education program 'Master of Science' (M.Sc.) in Public Health has been training graduates who are able to analyze and evaluate a wide range of health problems at the level of population groups and to develop and implement practical measures to solve them to evaluate.
Public health experts work and research in very different areas, functions and organizations. Therefore, the curriculum of the Düsseldorf continuing education course is designed to convey the methodological and practical basis of the subject. Study planning, data analysis, a critical reception of the literature, preparation and communication of research results, as well as the evaluation and planning of medical or preventive measures are skills that are universally used in healthcare. Based on international standards, the training in Düsseldorf therefore lays a broad scientific foundation on which those interested in public health can professionally further develop their own areas of focus.
The public health education oriented towards this goal is realized through interdisciplinary, practice- and research-oriented teaching, which is based on the principles of adult education and is systematically further developed on the basis of evaluations.
The continuing education course is integrated into the newly created structure of the Center for Health and Society of the Faculty of Medicine, which consists of the Institute for Medical Sociology, the Institute for Health Services Research and Health Economics, the Institute for General Medicine with the attached Cochrane Center, the Institute for Occupational, Social -

and environmental medicine, the working group on epidemiology of environmental influences in aging processes at the Institute for Environmental Medicine Research (IUF) and the Institute for Biometry and Epidemiology of the German Diabetes Center. This integration strengthens the research orientation of the continuing education course, which in this way is directly connected to numerous institutes and departments of the Düsseldorf Medical Faculty with a wide range of research activities and international cooperation. Examples include the Institute for Biometry and Epidemiology of the Leibniz Institute for Diabetes Research (German Diabetes Center) and the Leibniz Institute for Environmental Medicine Research. The program is designed in such a way that different professions can expand their qualification profile (see admission requirements). This also expressly applies to practicing physicians, who are important players in the design of health care and in health promotion."
"The teaching content and forms of the continuing education course are based on the unity of teaching and research and also convey methodological competence in addition to basic and specialist knowledge. In particular, it is about:

- Broad basic knowledge and orientation of the theoretical focus on current research developments in the subject areas
- In-depth methodological and strategic skills that enable independent scientific research
- Imparting interdisciplinary knowledge and the ability to integrate scientific approaches from different subject areas
- Involvement of students, if possible, in research and/or development projects as part of final theses" (From curriculum page)

Heinrich-Heine-Universität Düsseldorf AND Akademie für Öffentliches Gesundheitswesen: Public Health

"…Since 1991, the continuing education course at Heinrich Heine University in Düsseldorf has qualified experts who can identify, understand and solve health problems at population level.
Participants in the theoretical advanced training course "Public Health" of the Academy for Public Affairs health care can also complete the M.Sc. in Public Health/Public Health Care (M.Sc. PH/ÖG) from the Heinrich Heine University in Düsseldorf. This gives future specialists the opportunity to acquire additional skills in the field of public health and an internationally recognized university degree with a 60 CP variant of the course. While the focus of the qualification at the Academy for Public Health is on relevant skills for practical work in the public health service, the university focus is on academic qualification and scientific methodology". (From course pamphlet)

Jade Hochschule - Wilhelmshaven/Oldenburg/Elsfleth: Public Health

- …"Prevention and health promotion strategies
- Population Medicine and Planetary Health
- Research and methodological competence for the analysis and evaluation of e.g. B. epidemiological studies
- Participation and health literacy
- Qualification in impact and health research
- Qualification in health management and economics
- Consideration of health care and services from an ethical and economic point of view
- Dealing with scientific and practical questions and problems of the health care system
- Digital public health
- Evidence-based practice and evaluation of health care interventions"

(From Website)

FOM Hochschule für Oekonomie & Management - University of Applied Sciences: Public Health

"…In the Master's program "Public Health" you will acquire application-oriented specialist knowledge about the economic and social science structures and decision-making processes as well as the tasks of the players in the German and European health care system. They learn how diseases develop and how they affect individuals and society. In addition, you will deepen your knowledge in the field of health and medical sociology and become familiar with special legal aspects of the health economy and the management of health care companies. This enables you, among other things, to develop health-promoting measures and to optimize quality management systems."
(From Website)

Hochschule Fulda - University of Applied Sciences: Public Health

"…You will learn how to carry out research into health science subjects. Building on existing knowledge, the Research Methods module teaches you how to develop an appropriate research design, use quantitative and qualitative methods, present research outcomes and evaluate research. You acquire application-based research skills in the context of a research project, which will focus on the specific research task in small groups over a period of two semesters, and skills in the areas of epidemiology, writing papers for publication, economic evaluation and health technology assessment. In the second and third semesters, you develop your own focuses by choosing two compulsory elective modules and one of the international modules." (From website)
"…The Public Health degree is a research-oriented master's degree that offers university graduates with a health science focus the opportunity to qualify for population-related design tasks in the health care system.
Students develop a population-based perspective on health and disease. They learn to recognize and deal with socially determined health inequalities, to participate in the design of care structures and to work on strategies for global health problems." (From pamphlet)

Hochschule Fulda - University of Applied Sciences: Public Health Nutrition

"…The master's degree programme equips you to plan, implement, and assess interventions for the general population in prevention and health promotion in the field of public health nutrition.It also enables you to develop new approaches to the prevention and treatment of poor diet, provide advice on policy in the senior civil service, and assume design and decision-making roles in nationally and internationally-operating enterprises, political institutions, and in non-governmental organisations. The degree programme is based on biological/medical sciences, social sciences, and epidemiology. It recognises and demonstrates links between health, nutrition, and other health-related behaviour and identifies risk groups and prevention potential to prevent or treat lifestyle-induced disease with measures aimed at the general population." (From Website)
"…The course is intended for students who have completed their first university degree in the field of ecotrophology and related courses of study to enable to make decisions related to nutrition and health of the population, to advise others by preparing the relevant information, to plan, implement and evaluate appropriate measures, about nutritional and researching health science issues and taking responsibility for them further development of Public Health Nutrition. The course qualifies to work interdisciplinary" (from the module handbook)

"…The students develop a population-based perspective on health and illness; they learn socially to identify related health inequalities and to work on, in the design of supply structures to participate and to work on strategies for global health problems." (From Website)

Universität Bremen: Public Health - Gesundheitsförderung und Prävention

"…Students are enabled by the course:

- to systematically generate, formulate and justify research questions in the context of health promotion and prevention;
- to assign appropriate methods of empirical social research to the questions and to classify the range of the different research methods and study designs;
- apply different (quantitative and qualitative) methods of empirical social research;
- to ethically reflect on methodological decisions, to theoretically justify decision options and to transfer them to concrete fields of research and practice;
- Communicating, designing, implementing and evaluating practical projects in the context of prevention and (including communal) health promotion in a participatory manner and with a view to the respective target groups.
- Appropriately present their work through scientifically sound presentations, both orally and in writing." (From Website)

Universität Bremen: Public Health - Gesundheitsversorgung, -ökonomie und -management

"…The aim is to train experts in public health who can independently identify, analyze and solve complex problems in the areas of health care, economics and management so that they can contribute to an effective, efficient and fair supply system in different functions."
"…The aim of the course is to become experts in Public Health, who can independently identify, analyze and solve complex problems in the areas of health care, economics and management so that they can contribute to an effective, efficient and fair care system in different functions. The didactic concept corresponds to research-based learning. Students design their learning in an independently organized research process." (From Website)

Technische Universität Chemnitz: Public Health mit Schwerpunkt Prävention und Evaluation

"…The master's course imparts core competencies within the framework of a genuinely human and social science-oriented, quantitatively empirically oriented education with a focus on conceptual work for interventions in the health-related prevention area. This enables graduates to independently design intervention measures and to plan, implement and analyze empirical projects that can be used to evaluate health-promoting measures.
The emphasis on the area of evaluation also places specific methodological skills in the foreground." (From flyer)
"…The Master programme provides students with core competencies within the scope of a quantitative-empirical educational training that is genuinely oriented towards the subject of behavioural and social sciences and focusses on the conceptual work for interventions in the field of health-related prevention. This enables students to independently create concepts for intervention measures and to plan, execute and analyse empirical projects that can be utilised to evaluate measures of health promotion.

Emphasising the field of evaluation also gives priority to developing specific methodical expertise." (From website)

IU Internationale Hochschule: Public Health (120 ECTS)

"…In your distance learning Public Health M.Sc. you will acquire practical methodological skills in addition to well-founded theoretical knowledge. In the "Biostatistics and Demography" course, you will learn how to carry out static analyzes using software and thus determine valuable data on survival times and population structure. You will also dedicate yourself to action processes and case studies from the everyday life of public health professionals and thus prepare yourself comprehensively for your future activities." (From Website)

IU Internationale Hochschule: Public Health (60 ECTS)

"…In your distance learning Public Health M.Sc. you will acquire practical methodological skills in addition to well-founded theoretical knowledge. In the "Biostatistics and Demography" course, you will learn how to carry out static analyzes using software and thus determine valuable data on survival times and population structure. You will also dedicate yourself to action processes and case studies from the everyday life of public health professionals and thus prepare yourself comprehensively for your future activities." (From Website)

Technische Hochschule Mittelhessen – THM: Public Health

"…The Master's degree in Public Health focuses on the skills required to analyze the social, political and health development of the population and to design and evaluate new forms of care. The main objective is to analyze and improve health care and thus population health. The course offers the opportunity to focus on the sector of health services research or rehabilitation.
The study program builds on a completed bachelor's or diploma degree in the field of medical management or a related course in the health sector. The Master's degree enables you to carry out scientific research and take on strategic and managerial tasks in the field of healthcare."
"…The Master's degree in Public Health focuses on the meta-level of the healthcare industry and imparts the relevant methodological and technical skills from the core areas of public health research defined by the World Health Organization (WHO). The course also addresses the rehabilitation sector, which has so far received little attention but is becoming increasingly important due to demographic change, and pays particular attention to health services research. This possible focus clearly distinguishes this course from similar courses. You will be enabled to analyze the changing social circumstances of a population as well as environmental factors and their influence on the state of health or the ability of people to work and to derive consequences from them or to define and implement the resulting new requirements for the health system and politics. Students can analyze health data and, for example, identify widespread diseases or analyze the spread of epidemics or, ideally, prevent them with suitable concepts.
By interlinking various specialist areas such as health services research, rehabilitation research, social and environmental medicine, digital technologies and communication and by strengthening your social skills, you will be optimally prepared for the diverse tasks in research and practice.

The MSc Public Health allows students to study independently and individually according to their personal inclinations. This is achieved, among other things, by the freedom of choice of the compulsory elective modules." (From Website)

Hochschule für Angewandte Wissenschaften Hamburg: Public Health

"…The Master of Public Health course aims to equip you with the awareness, knowledge and skills required to change the trajectory of these deep-rooted and emerging health issues."
"…There is growing national and international demand for highly trained professionals within the changing field of public health. This Master of Public Health degree course is ideally suited to professionals aiming to attain academic knowledge and skills in evidence-based practice, empirical research and the implementation of research findings as well as in designing and improving public health policies and practices at the regional, national and global levels."
"…The Master of Public Health students acquire the ability to deal with the conditions for health and the management of illness rooted in the natural, technical and social environment of human beings. The students know the essential factors that influence the health situation of a population, as well as the decisive role of the health care system, the supply structures, the financing of health care services, political and social conditions and also the health behaviour of the population. The students will be enabled to develop approaches to solutions for improving the overall health situation in society." (From module handbook)
"…You can apply health science theories and methods to population level
• the political and economic framework of a population group to analyze health-related
• examine the systems of health care,
• to consider the interactions between people and their environment,
• the social structures and health-related behaviours of society explore,
• to implement health science findings in society as a whole.
One of their later tasks is to have an effect on social relations to influence and change. This can be done by taking on management functions in the supply area, cooperation at the political level or through research and evaluation and dissemination of health science concepts". (From examination regulations)

Ludwig-Maximilians-Universität München: Public Health

…The course deepens subjects that play an important and increasingly important role in healthcare. It imparts theoretical basics, practical skills and competencies for tasks in the field of health sciences, health care and population medicine in research, education, management, administration, politics and industry. (From Website)

Medizinische Hochschule Hannover (MHH): Bevölkerungsmedizin und Gesundheitswesen (Public Health)

"…The course imparts analytical-methodical and intervention-related content. It builds on the experiences of future graduates from previous studies and relevant professional activity in the healthcare sector.
Graduates gain an in-depth understanding of the German healthcare system, also in comparison to international systems, analyze the different actors and their different interests get to know current control and financing models, can analyze and change management processes of selected institutions, can apply concepts of quality management, are able to consider cost issues and carry out health economic studies.

You can also use epidemiological and statistical methods safely

- analyzing statements about avoidable and unavoidable morbidity and mortality of the population
- critically evaluate scientific investigations, carry them out and analyze them yourself
- assess the importance and concepts of health promotion, prevention, treatment and rehabilitation and develop, implement and evaluate their own concepts
- implement strategies for health education and health education
- collect and represent the interests of the users of the system." (From Website)

Universität Siegen: Digital Public Health

"…The Digital Public Health master's degree is intended to impart basic specialist knowledge in the analytical and methodological area in connection with application and implementation-oriented knowledge...Their interdisciplinary knowledge and methodological skills enable them to work as team members or in the management of projects in research, with payers, in politics or in the private sector. In doing so, they take a holistic perspective and shape the digital transformation of our living environments in a health-oriented way." (From website)
"What can I do after graduation?:
• Conception, introduction and evaluation needs-based and effective digital interventions in living environments
• Participation in and management of teams in the public health service, in research institutes, companies, health insurance, politics, and much more". (From presentation linked on website)

Technische Hochschule Deggendorf: Global Public Health

"…Skills:
The graduates have the competence
● lead research, development and innovation projects and they master the methods of research and development work.
● new through the combination of expertise from different fields create information and improve working methods.
● to develop service-oriented, sustainable and profitable solutions.
● to communicate clearly in their work and in the development of measures internationally in English and another language in word and to act in writing.
● think and act entrepreneurially and formulate strategies.
● implement theoretical knowledge in a practical and solution-oriented manner.
● the importance of planning and strategic thinking as a resource for the building a global that is both professional and close to the citizen understand public health promotion.
● secure evidence-based information and advanced solutions to apply for better health and well-being.
● manage change processes necessary for an advanced global public health are necessary.
● Assess sustainable global public health in accordance with Human Security and One Health principles in a multidisciplinary network and to develop the variety of methods used to assess the health of the population understand and apply them.
Qualification goals M-GPH
Technical University Deggendorf / EM 7
● the health and welfare of all species living on earth improve and defend.
● Networks and partnerships in global public health evaluate, use and develop them.
● to organize themselves as well as team skills and leadership skills of interdisciplinary cooperation.

● their actions on ethical, ecological, social and economic reflecting on and aligning requirements." (Found in a document titled "Qualifikationsziele Joint Master Global Public Health" only available on the German version of the homepage)

Leuphana Universität Lüneburg: Prävention und Gesundheitsförderung

"…Aims and perspectives: the master of public health makes you fit for a healthy future.
The Master's degree in Prevention and Health Promotion opens up new professional opportunities for you and makes you sought-after experts in the healthcare sector.
They have a wide variety of skills

- the scientific analysis,
- the strategic planning
- and the implementation and evaluation of health-related projects.

You are able

- to develop health-promoting programs for practice, taking into account economic, legal and ethical aspects,
- put into practice the principle "promote health, avoid disease",
- to support and act preventively." (From Website)

APOLLON Hochschule der Gesundheitswirtschaft: Public health - Umwelt & Gesundheit

"…Which key qualifications are taught?
The course provides you with all the skills and abilities to analyze conditions for health and illness. In doing so, you consistently include the environment with its pathogenic and salutogenic aspects. On this basis, you will develop evidence-based prevention approaches involving various disciplines, which you can also evaluate in terms of effectiveness.
Specifically, you acquire:

- Knowledge of national and international health systems
- Sound knowledge of public health and global public health
- Specific methodological skills for health economic evaluation, evidence-based medicine and health technology assessment as well as medical writing
- In-depth knowledge of climate change and health, sustainability and sustainable development
- Knowledge of health hazards and health protection related to the environment
- In-depth insights into health and environmental policy
- Health and environmental psychological know-how" (From Website)

Competencies doctoral programs

Heinrich-Heine-Universität Düsseldorf - Dr. PH (Public Health)

NA

Medizinische Hochschule Hannover – Dr. Public Health

”…Public health research is interdisciplinary and takes into account a variety of individual disciplines (e.g. sociology, psychology, medicine, epidemiology and economics). Recently,

the COVID-19 pandemic in particular has highlighted the relevance of public health research and practice for population health and the health care system.” (From Website)

(1) “…1. On the basis of the following provisions, Hannover Medical School (MHH) awards the degree of Dr. Public Health (abbreviated and used in the following as: Dr. PH) for scientific achievements in research areas within the framework of the subjects represented by it. 2. The doctorate serves as proof of the ability to conduct in-depth independent scientific work. Proof is provided by the submission of a scientific paper (dissertation) and an oral examination (disputation).”

Research studies at the Hannover Medical School (MHH) for the purpose of obtaining a PhD or Dr. rer. nat. degree (hereinafter referred to as PhD studies) shall facilitate postgraduate training with a focus on specific research projects with a view to enabling the candidate to do in-depth scientific work on his or her own and to provide him or her with additional professional qualifications for future assignments in research or related areas of work. PhD studies shall foster the development of outstandingly gifted up-and-coming academics." (From Doctoral degree regulations)

Rheinische Friedrich-Wilhelms-Universität Bonn - Doctorate PhD (Public health, epidemiogy, health services research)

§4 "The qualification phase is research oriented. Doctoral students should acquire a well-founded understanding of academic problems, in-depth expert knowledge and the ability to undertake interdisciplinary work."
§6 "The doctoral thesis must be an academic work that represents an advancement in academic knowledge and demonstrates the ability to undertake independent academic work and present the results in an appropriate manner." (From Doctoral degree regulations)

Charité – Universitätsmedizin - Health Data Sciences

"…The PhD Program in Health Data Sciences at the Charité is aimed at qualified young scientists interested in:

- deepening their methodological knowledge in the fields of biostatistics, epidemiology, public health, meta-research, population health science and medical informatics.
- further expanding their competence in research and teaching."

"…Health Data Sciences (HDS) is an emerging scientific discipline, fusing the strengths of multiple research fields including biostatistics, epidemiology, meta-research, public health, population health science and medical informatics. HDS employs a synergistic approach to provide comprehensive, evidence-based solutions to complex, real-world health problems using various data resources.
By employing innovative analytical tools, HDS methodology provides insight both into patterns within data as well as possible underlying causal structures. Its comprehensive modeling approaches combine critical and conceptual reasoning with the latest advances in computer science to ensure accurate analysis and interpretation of data as well as effective communication of reliable conclusions for health promotions."
"…The structured PhD program in Health Data Sciences at the Charité - Universitätsmedizin Berlin offers both German and international students an interdisciplinary educational environment:
• to deepen their methodological knowledge in the fields of biostatistics, epidemiology, meta-research and/or population health science and
• to expand their competencies in both research and teaching.
The ultimate goal of the doctoral program is to prepare students for careers in universities and research institutions through excellence in research, methodology and teaching." (From program flyer)

"…The doctoral program in Health Data Sciences requires all graduates to demonstrate advanced methodological skills in Biostatistics, Epidemiology, Meta Research or Population Health Science....The doctoral program in Health Data Sciences requires all graduates to develop teaching skills for potential careers in academia" (From Doctoral degree regulations)

LMU – PhD Medical Research

"…A doctorate in our internationally oriented, English speaking Ph.D. program enables graduates from different scientific backgrounds to sharpen their competence in epidemiology and the public health sector. Multidisciplinarity on a high level helps to train young scientists in epidemiological approaches and public health questions in their future professional life... Our students receive individually tailored training in research methodology as well as in transferable skills" (From program flyer)
"…research and training opportunities in the following fields:
• Clinical, Molecular and Genetic Epidemiology
• Clinical Trials and Translational Medicine
• Preventive Geriatrics
• Genomic and Molecular Medicine
• Preventive Geriatrics
• Digital Health
• Clinical and Evidence-based Prevention
• Health Services Research
• Health Economics and
• Evidence-based Public Health" (From the main page)

University of Tübingen - PhD Program in Experimental Medicine

"…To provide excellent young researchers in medicine and the life sciences with advanced professional qualifications for a career in research."
"(1) The PhD program Experimental Medicine at the Faculty of Medicine Tübingen provides project-oriented postgraduate training in basic and applied medical research. It is designed to promote highly talented young scientists. The goal of the program is to enable students to carry out independent scientific work and to provide them with advanced professional qualifications for jobs in research or related areas." (From Section 1 of Study regulations)

Helmholtz Centre for Infection Research AND Hannover medical school AND Hannover biomedical research school - PhD Programme Epidemiology

"…During the course of the programme, PhD fellows are being prepared to conduct independent epidemiological and public health relevant research. After successful completion, the fellows can enter career paths in academia, research and science, national health institutions, ministries, health insurances, international organizations or other non-governmental organizations." (From Website)

Universität Bielefeld – Dr. PH

"…acquire the knowledge and skills to work independently and systematically with suitable methods on scientific problems from the fields of health sciences and the international scientific field of public health, expands the theoretical knowledge base of the doctoral candidates and their methodological competence and enables them to further develop and

apply new theoretical, methodological and practical knowledge in the health sector in order to produce a high quality and original dissertation.
The programme enables graduates to collaborate with academics and practitioners in solving health problems and to contribute to addressing political, ethical and social problems in health care.
The degree programme prepares graduates to take up junior academic positions in universities and research institutions and/or to take up leading professional positions in public or private institutions in health policy and health care.
Tasks range from quality assurance of care to implementing programmes for special populations or identifying and responding to the care needs of people in different disease situations, the skills to develop new study programmes, to recruit, plan and carry out research projects, as well as the skills to publish the results of health science projects and disseminate them to the professional public." (From Website)
