# Supplementary material for: A systematic mapping of public health master’s and structured doctoral programs in Germany
Source: BMC Med Educ. 2024 Aug 13;24:872. doi: 10.1186/s12909-024-05855-8 (PMC11323405; doi:10.1186/s12909-024-05855-8)
Supplement: Supplementary file 9 — Additional file 9. (Extracted data on work fields suggested by public health master’s and doctoral programs) [file 12909_2024_5855_MOESM9_ESM.pdf]

Additional File 9 – Data extraction work fields for public health master’s and doctoral programs

Work fields for master’s programs

University Bielefeld: Public Health

"…Fields of activity for our graduates are, for example: research and teaching at universities, project work and management as well as scientific support in public (health) administration (e.g. RKI), work as a consultant for social insurance (health, nursing and pension funds).), project work and management as well as scientific support in company health management in commercial companies, institutions and facilities of the social sector and speaker* at international organizations (e.g. NGO, WHO).
Open for graduates of the Public Health master's degree numerous entry opportunities in different professional fields. For example, they can in the following institutions work: research institutions, health and nursing care insurance companies, Associations, clubs and foundations, offices and authorities, hospitals or advice centers as well as in the private sector. International courses prepare the students Fields of activity with international references or abroad.
In addition to a direct career path, the Master's degree also offers graduates the entry requirements, to apply for the faculty's doctoral program in Public Health to apply for health sciences." (From website)

Charité AND Technical University of Berlin AND Alice Salomon Hochschule Berlin: Public Health

"…A master's degree in public health serves to deepen the content and expertise and specialize for activities in the healthcare sector.
Graduates of the BSPH can opt for a research-oriented orientation and are equipped for science-related work.
It can also be studied in a more application-oriented way, which enables a wide range of uses in health administration and health policy, planning and management of and in care facilities, supporting organizations and associations in the health and social sector." (From ASH)
"A master's degree in public health serves to deepen the content and expertise and specialize for activities in the healthcare sector.
Graduates of the BSPH can opt for a research-oriented orientation and are equipped for science-related work.
It can also be studied in a more application-oriented way, which enables a wide range of uses in health administration and health policy, planning and management of and in care facilities, supporting organizations and associations in the health and social sector." (From Charité website)

Technical University of Dresden: Gesundheitswissenschaften - Public Health

"…Possible professional fields, e.g.:
— Research and educational institutions
— Project and program management in the public sector
service (e.g. health authorities, statistical
state offices etc.)
— health insurance
— Associations and institutions in the healthcare sector

— pharmaceutical industry
— International organizations (e.g. WHO etc.)" (From website)

APOLLON Hochschule der Gesundheitswirtschaft: Public Health - Prevention and Mental Health

"…Graduates can all fields of work in which both theoretical and practical knowledge about the health development of specific population groups in different health environments is required. For example, you will learn to analyze the mental health of children and young people in the school setting with a focus on mobbing and bullying, to develop modern health-promoting strategies or to check and improve the effectiveness of preventive interventions.
Therefore, the course not only qualifies you for responsible positions in public and private health institutions, but also for exciting positions in private companies. Here you can, for example, take on tasks in company health management, in psychological risk assessment, behavioural and relationship prevention to mental health at work or burnout prevention." (From website)

Heinrich-Heine-Universität Düsseldorf: Public Health → 120 ECTS

"…Although there is no clearly defined job description for "public health" experts, the public health qualification is well established and a wide range of professional activities is opening up, and in some areas it is still dynamically growing.
"A formal public health qualification (MPH) is required or desired for some job advertisements (e.g. for physicians in the field of international organizations such as the WHO).
The experience of the public health course in Düsseldorf, which has been in existence for more than 20 years, confirms a diverse professional field of activity. Graduate surveys show again and again that numerous graduates have taken up a new job for which the degree was an essential prerequisite. The course was also used for professional training and personal skills development. The most important professional fields for graduates are:

- Scientific work at universities, technical colleges, public research institutions
- Managerial activities in patient care (inpatient, outpatient, company medical service)
- Activities in self-governing organizations (regional, state and federal level; medical and health insurance organizations)
- Public Health Service
- Worked in international organizations and in development aid
- Structure relaunch public health
- leadership roles in healthcare organizations
- Worked in management consultancies and in private industry". (From website)

Heinrich-Heine-Universität Düsseldorf AND Akademie für Öffentliches Gesundheitswesen:Public Health → 60 ECTS

"…Since 1991, the continuing education course at the Heinrich Heine University in Düsseldorf has qualified experts in the healthcare sector to analyze health problems at population level and to implement solution concepts.
Participants in the theoretical advanced training course "Public Health Care" at the Academy for Public Health Care can also complete the M.Sc. Public Health/Public Health Service (M.Sc. PH/ÖG) from the Heinrich Heine University in Düsseldorf.
This gives future specialists the opportunity to acquire additional skills in the field of public health and an internationally recognized university degree with a 60 credit point variant of the course." (From AÖGW website)

Jade Hochschule - Wilhelmshaven/Oldenburg/Elsfleth: Public Health

"…The course provides graduates with the necessary skills and Competences to take on specialist and managerial positions in the healthcare sector and in the healthcare industry. With that you are experts, such as:
• in company health and quality management
• at utilities, such as the Health authorities, health insurance companies as well Associations of Statutory Health Insurance Physicians
• at supporting organizations, such as associations of the health and social services
• at vocational academies, in research and teaching at universities or as doctoral candidates". (From flyer)
"The master's degree not only promises excellent preparation for your career, but also ensures good application possibilities of what you have learned in your job. Now you have a good chance of improving your already qualified position in the health sector, occupying new professional fields and filling managerial positions.
* supply facilities, such as health authorities, health insurance companies and associations of panel doctors
* supporting organizations, such as associations in the health and social sectors
*research and teaching at universities
* training sector
* the corporate health sector
* the management and internal coordination of clinics and public institutions
* health and quality management
*at vocational academies and technical schools". (From website)

FOM Hochschule für Oekonomie & Management - University of Applied Sciences: Public Health

"…The course qualifies you for the following tasks

- Management and coordination of projects for health promotion and disease prevention with the aim of increasing and optimizing the quality of life and care
- Conception, implementation and evaluation of needs-based health-promoting structures and measures, taking into account economic, legal, social and ethical requirements
- Analysis of the determinants of health and disease
- Development and assessment of scientific solutions for the further development of the health system
- Development and implementation of quality management systems in healthcare". (From website)

Hochschule Fulda - University of Applied Sciences: Public Health

"…University graduates from different disciplines with a professional or academic focus on health care issues have the opportunity to qualify for executive positions in health care. Public health practice requires the ability to think and act in a population-based way and not to focus exclusively on optimising the economic results of health service providers. If you would like to assume responsibility for designing the health systems in a national or international context or wish to research and teach these subjects, this is the right programme for you."
"…An academic career path is usually pursued through work in externally funded projects at universities or a doctoral degree. Research-oriented activities, however, are also possible at

non-university institutes such as the Robert Koch Institute or in the area of health technology management.
In a domestic context, you might take up a strategic and conceptual position in public administrations on a municipal, local or national level, in the area of social insurance or in quality assurance institutions in the health system. Political consultancy and association work are also possible options.
The Master's degree in Public Health is often a useful stepping stone for advancement to senior positions within care structures.
For a position in development cooperation, experience abroad and proficiency in English and a second international language are usually additional recruitment requirements alongside a Master's degree in Public Health."
"…Once you have graduated, you may choose to pursue a doctoral degree, or you might take up a position in health science research or teaching; you also might work in health reporting at the communal, state, or federal level.
You might work in an institution involved in quality assurance in the health system, where you would contribute to producing systematic reviews or Health Technology Assessments.
Another option is consulting within the health system: If you have gained relevant experience abroad, this might be in developmental collaborations, or in internationally operating organisations, but also in government departments, in other state authorities or agencies, in associations, or in the administration of health care institutions.
You may contribute to new concepts for care structures or for health promotion, or you might fill executive or managerial positions at pension providers, or you might be responsible for campaigns and projects." (From website)

Hochschule Fulda - University of Applied Sciences: Public Health Nutrition

"…The master’s degree qualifies you to

- Identifying nutritional problems nationally and in different regions of the world using epidemiological methods, describing their causes and developing solutions for potential nutritional problems.
- to plan, implement and evaluate population-based health promotion and prevention interventions in the field of public health nutrition.
- to develop new coping approaches in the prevention of malnutrition and to take on tasks in political consulting in the public sector.
- To take on design and decision-making tasks in national and international companies.
- to work in political institutions and in non-governmental organizations.
- to work in an interdisciplinary manner, i.e. to cooperate professionally with specialists in the social sciences, in health education and health promotion.

Another path taken by many of our graduates is entry into research, e.g. B. via a doctoral position. It is possible to do a doctorate at the Fulda University of Applied Sciences after completing your studies."
"…The master’s degree programme equips you to plan, implement, and assess interventions for the general population in prevention and health promotion in the field of public health nutrition.It also enables you to develop new approaches to the prevention and treatment of poor diet, provide advice on policy in the senior civil service, and assume design and decision-making roles in nationally and internationally-operating enterprises, political institutions, and in non-governmental organisations. A further option, taken by many of our graduates, is to take up a career in research via a doctoral position." (From website)

Universität Bremen: Public Health - Gesundheitsförderung und Prävention

"…Possible fields of activity for graduates are, for example, in the areas of research and teaching, health promotion and prevention in different settings (company/authorities, community/neighborhood, hospital, school, day care center, etc.), planning of population or group-related measures (in the context from health insurance companies, specialist authorities of the Federal Ministry of Health or corresponding state ministries, associations and clubs in the health sector); health advice and health education; health-related public relations and communication etc.". (From website)

Universität Bremen: Public Health - Gesundheitsversorgung, -ökonomie und -management

"…Successful graduates are prepared to develop science-based solutions for complex tasks and to participate in the design of health care systems at national and international level. Possible fields of activity are in research (universities, free institutes) in the scientific foundation of the health system (e.g. G-BA, IQWiG, associations, authorities), care planning (e.g. health insurance companies, associations), health care policy (e.g. associations of doctors, health insurance companies, industry), political advice (e.g. for foundations, political parties), contract management (e.g. for health insurance companies, independent service providers), for non-profit associations or international organizations.
Courses offered for the career goal of school
No courses offered for teaching at public schools
Opportunities for subsequent further qualification
The degree entitles you to take a doctorate." (From website)

Technische Universität Chemnitz: Public Health mit Schwerpunkt Prävention und Evaluation

"…Beyond research, students participating in this study programme receive a first-rate preparation for management positions in the fields of

- Market research
- Pharmaceuticals
- Health insurances
- The Public Health Service, i.e. local health authorities, State Offices for Health as well as federal organisations and institutes (Robert Koch Institute, Paul Ehrlich Institute, Federal Centre for Health Education, etc.)
- Private-sector healthcare providers
- Medical wholesale and retail sector." (From website)

IU Internationale Hochschule: Public Health (120)

"…Intelligent strategies in healthcare are more important than ever and your career prospects are extremely promising. After successfully completing your public health distance, a wide range of tasks in health policy, health and social affairs as well as activities in the field of health communication and prevention, health promotion or the pharmaceutical industry await you. For example, you work as...
*Program management in the Federal Ministry
As head of a program related to health in a federal ministry, you use your extensive knowledge to provide technical and strategic advice to decision-makers. You put together your own project team of promising talents and make sure that all processes are designed as efficiently as possible. At the same time, you will build up an international network of politicians and interdisciplinary professionals.

* Senior associate in the consulting firm
The physical and mental health of employees is important in many companies. Professional advice is required, especially in times when the number of people affected by burnout is increasing drastically. In the position of senior associate, you will take care of project management, technical advice and the implementation of projects for your customers. With your Master Public Health distance learning you can also take on team leadership and management positions.
* Scientific power for health
Which measures efficiently promote the health of different population groups? As a scientific force in health promotion, you devote yourself to questions like this one. You develop, implement and evaluate programs designed to improve the health and quality of life of your target group. You then publish the results in specialist journals or present them at congresses. Your tasks may also include the training of employees and cooperating companies." (From website)

IU Internationale Hochschule: Public Health (60)

"…With your master's degree, you will develop further – professionally and personally. In addition to new knowledge, you will acquire key skills that will qualify you for sought-after specialist and management positions. You also benefit from the chance of a lucrative salary increase."
"…focus on your career" - "you choose one of nine elective modules, such as "Gender, Participation and Health", "Infectious Diseases and Global Health" or "Innovation in Pharma and Medical Technology". (From website)

Technische Hochschule Mittelhessen – THM: Public Health

"…The Master's degree qualifies for scientific research and for strategy and management tasks in the field of health care. Graduates are qualified for higher management tasks as well as for health science leadership and consulting in various public and private institutions of the health care system such as authorities, research institutes, international health organizations, associations, health insurance companies as well as hospitals, rehabilitation or care facilities." (From website)

Hochschule für Angewandte Wissenschaften Hamburg: Public Health

"…As a public health professional, you will be qualified to take up positions in the following areas:

- International health organisations and NGOs
- Health promotion organisations
- Health management in hospitals and public administration
- Health research and surveillance institutions
- Health insurers and pharmaceutical businesses". (From website)

Ludwig-Maximilians-Universität München: Public Health

"…Master of Science Public Health: With this training, graduates qualify for professional fields in the following areas, for example:
educational and research institutions

- Project and program management in authorities (health authorities, state statistical offices, federal offices such as statistics, radiation protection or risk assessment)
- healthcare

- health insurances
- Associations and institutions in the field of health economics and health system research
- pharmaceutical and food industry
- business consulting
- International organizations (e.g. WHO, UNDP, UNEP)"

"…A master's degree in Public Health opens up a variety of career opportunities. Students gain theoretical knowledge, practical skills and key competencies that qualify them to work in the broad field of Public Health. Upon graduation, many alumni continue working in research, often to pursue a PhD degree. Another very popular career path among our alumni is provided by the job openings in Public Health institutions at the local, regional, national or international level. Many of our alumni also take up employment in health insurance companies, in the field of workplace health promotion or in the pharmaceutical sector." (From info brochure)

Medizinische Hochschule Hannover (MHH): Bevölkerungsmedizin und Gesundheitswesen (Public Health)

"…Potential employers of public health graduates:
* Federal institutions
* Institutions of the governments and administrations of the countries
* Social Security Agency
* Social partners and umbrella organizations of the German economy
* Institutions of outpatient and inpatient health and old age care
* Educational institutions
* Business enterprise
* Occupational health and safety centers
* Private institutes for research, planning and consulting
* Relevant university and technical college departments for research and teaching
* International health organizations" (From additional document linked on website)

Universität Siegen: Digital Public Health

"…The Digital Public Health master's degree is intended to impart basic specialist knowledge in the analytical and methodological area in connection with application and implementation-oriented knowledge. This opens up numerous entry opportunities for graduates in various professional fields such as research institutions, health and nursing care insurance companies, social welfare associations, clubs and foundations, public health departments and authorities, hospitals or advice centers as well as in the private sector. Her special expertise at the interface between health, digitization and society is in great demand in order to design the processes of digital transformation in our living environments in an evidence-based and needs-based manner. you conceptualize

Thanks to their interdisciplinary knowledge and their methodological skills, they are able to work as team members or to manage projects in research, for payers, in politics or in the private sector. They take a holistic perspective and shape the digital transformation of our living environments in a health-friendly manner.

The master's degree offers graduates not only a direct career path but also the admission requirements to apply for a doctoral degree in the field of public health." (From website)

Technische Hochschule Deggendorf: Global Public Health

"…After successfully completing this postgraduate degree, expect to build your career in sectors including:

- Governments
- NGO’s
- Consultancies
- International organisations (e.g. UNDP, UNFPA, WHO)
- Healthcare management
- Health promotion
- Human resource management
- Administration
- Marketing and controlling
- Occupational healthcare". (From website)

Leuphana Universität Lüneburg: Prävention und Gesundheitsförderung

"…The Master's degree in Prevention and Health Promotion is aimed at specialists and executives from the health, social and educational sectors and the private sector who would like to acquire both practical specialist knowledge and key personal skills and want to increase their chances in the growth industry of "prevention and health promotion". The master's degree in the field of health management also qualifies you for specialist and managerial tasks with scientific competence." (From website)

APOLLON Hochschule der Gesundheitswirtschaft: Public health - Umwelt & Gesundheit

"…Industries:
-Health insurance companies/social security institutions
-research facilities/scientific institutes
- Professional societies/associations/politics
-public health service/city administration
- Engineering offices/urban planning
- welfare and social organizations
position as:
-consultant municipal health promotion
- Self-employed environmental and health consultant
-Specialist for prevention and health promotion
- scientific staff
-Team leader supply and health management
- project leader research
- consultant for health-related urban development".
"…Whether they are companies, clinics, health insurance companies, federal, state and municipal institutions or research: the interdisciplinary management of the relationship between environment and health plays a key role at all levels. We are looking for prevention experts who also take relevant environmental influences into account. People with environmentally oriented know-how are needed to analyze the health protection of specific population groups (e.g. older people) or in different health environments (e.g. municipalities, companies, old people's homes, kindergartens etc.), to develop suitable strategies and to Measure and improve the effectiveness of preventive interventions. As a master's graduate, you have a brand-new skills profile that qualifies you for such a responsible job."

Work fields for doctoral programs

Heinrich-Heine-Universität Düsseldorf - Dr. PH (Public Health) – no info

Medizinische Hochschule Hannover – Dr. Public Health – no info

Rheinische Friedrich-Wilhelms-Universität Bonn - Doctorate PhD (Public health, epidemiogy, health services research) – no info

Charité – Universitätsmedizin - Health Data Sciences

"…The ultimate goal of the doctoral program is to prepare students for careers in universities and research institutions through excellence in research, methodology and teaching" (From program flyer)
"…The program places particular emphasis on preparing students for future careers at universities, non-university research institutions, and other scientific public or private institutions in the fields of biostatistics, epidemiology, meta-research, population health science, public health and medical informatics" (From second link webpage)

LMU – PhD Medical Research

"…Research and training opportunities in the following fields:
• Clinical, Molecular and Genetic Epidemiology
• Clinical Trials and Translational Medicine
• Preventive Geriatrics
• Genomic and Molecular Medicine
• Preventive Geriatrics
• Digital Health
• Clinical and Evidence-based Prevention
• Health Services Research
• Health Economics and
• Evidence-based Public Health". (From website)

University of Tübingen - PhD Program in Experimental Medicine

"…The target group are graduates of medical courses (human medicine, dentistry) and related subjects such as molecular medicine medical technology, natural sciences, who are interested in an intensive scientific education and would like to prepare for work in the field of medical research and basic research...
VISION
To strengthen the link between science and applied medicine by preparing and equipping excellent students for a career in life science
OBJECTIVES
To provide excellent young researchers in medicine and the life sciences with advanced professional qualifications for a career in research". (From website)

Helmholtz Centre for Infection Research AND Hannover medical school AND Hannover biomedical research school - PhD Programme Epidemiology

"…After successful completion, the fellows can enter career paths in academia, research and science, national health institutions, ministries, health insurances, international organizations or other non-governmental organizations". (From website)

Universität Bielefeld – Dr. PH

"…Doctoral training is viewed as a "hinge" between the universities' educational and research mandates. It delivers a degree that is recognized worldwide in academia as well as in business, industry and government.

In recent years in Germany it has again become a major focus of educational policy interest and the character of doctorates has changed as a result. Although these still serve as an academic qualification today, their importance as proof of professionally relevant skills at the highest level has increased significantly. More than 300 health scientists nationwide have the academic degree of Doctor of Public Health (DrPH). Most of them “freely did their doctorate” in a conventional way, mostly as lone fighters and in a traditional two-person constellation doctoral supervisor. Initial studies of the experiences of these successfully completed doctoral students at the School of Public Health in Bielefeld clearly show that the majority of health scientists with a doctorate hold management and leadership positions in academic institutions and in health policy as well as in typical areas of health and social services hold. It shows, that Doctorates are increasingly becoming a prerequisite for certain professional positions, and not only in the professional field of science and research.

A large number of graduates with the doctoral hat practice managerial tasks in authorities, ministries and public institutions, in research institutes and agencies as well as in management positions in commercial healthcare facilities such as the public sector health service and hospitals. Your responsibilities extend from Quality assurance of care up to the implementation of programs for special population groups or the determination and answering of the care needs of people in different disease situations. The structured doctoral program addresses all of these areas of competence. Many doctoral students prepare in good time for teaching at universities, technical colleges and further education academies and for a career as a university lecturer in order to set the course for young scientists in public health. You need the skills to develop new ones study programs, for the recruitment, planning and implementation of research projects as well as the ability to publish the results of the health science projects and their dissemination to the professional public. The Bielefeld doctoral course meets these requirements in order to promote and promote the strengthening of the doctorate as a key role for a junior scientific career in the health sciences." (From info brochure - career opportunities for graduates).
